# Chemotherapy‐Mediated Induction of PD‐L1 via SEI1 Facilitates Myeloma Immune Evasion

**DOI:** 10.1002/advs.202411082

**Published:** 2025-03-26

**Authors:** Rui Chen, Zongwei Li, Zhihong Fang, Zou Li, Daoyan Yang, Yuan Li, Shurong Liu, Zhiqiang Liu, Rui Liu, Huan Liu

**Affiliations:** ^1^ Cancer Research Center School of Medicine Xiamen University Xiamen 361102 China; ^2^ School of Life Sciences Anhui Medical University Hefei Anhui 230032 China; ^3^ Department of Hematology The First Affiliated Hospital of Xiamen University and Institute of Hematology School of Medicine Xiamen University Xiamen 361102 China; ^4^ Department of Hematology Key Laboratory of Xiamen for Diagnosis and Treatment of Hematological Malignancy Xiamen 361102 China; ^5^ Shandong Provincial Key Laboratory of Radiation Oncology Shandong Cancer Hospital and Institute Shandong First Medical University and Shandong Academy of Medical Sciences Jinan 250117 China; ^6^ Shenzhen Research Institute of Xiamen University Shenzhen Guangdong 518057 China

**Keywords:** chemotherapy, immune evasion, multiple myeloma, PD‐L1, SEI1

## Abstract

Multiple myeloma (MM) is a plasma cell‐derived malignancy. While immune checkpoint blockade immunotherapy has advanced myeloma treatment, chemotherapy remains the primary therapy. How chemotherapy interacts with immune checkpoint expression and impacts immunotherapy efficacy remains unclear. Here it is discovered that chemotherapeutic drugs induce DNA damage and activate the cyclic guanosine monophosphate (GMP)–adenosine monophosphate (AMP) synthase (cGAS)/stimulator of interferon genes (STING) signaling pathway. This activation promotes phosphorylation of the interferon regulatory factor 7 (IRF7), which binds to the promoter region of SERTA‐containing domain 1 (*SERTAD1*, also called *SEI1*) gene to enhance its transcription. The SEI1 directly interacts with the enhancer factors CREB‐binding protein (CBP)/p300 and RNA polymerase II (pol II)‐associated factor 1 (PAF1) complex, promoting transcriptional activity and leading to upregulation of programmed death ligand‐1 (PD‐L1) and immune escape in myeloma. Both in vitro and in vivo experiments demonstrate that treating myeloma cells with PD‐L1 antibodies post‐chemotherapy significantly enhances the killing efficiency of activated T cells, compared to sequential treatment with chemotherapy and PD‐L1 antibodies. This research not only uncovers a pivotal regulatory mechanism of PD‐L1 upregulation but also provides a compelling rationale for the integration of chemotherapy and immunotherapy in myeloma treatment.

## Graphical abstract

Chemotherapy induces DNA damage, activating cyclic guanosine monophosphate (GMP)‐adenosine monophosphate (AMP) synthase (cGAS)/stimulator of interferon genes (STING), which phosphorylates interferon regulatory factor 7 (IRF7) to drive SERTA‐containing domain 1 (SEI1) transcription. SEI1 recruits CREB‐binding protein (CBP)/p300 and polymerase II‐associated factor 1 (PAF1) complex, upregulating programmed death ligand‐1 (PD‐L1) for immune escape. Post‐chemotherapy PD‐L1 blockade enhances T‐cell cytotoxicity, supporting combined chemo‐immunotherapy.

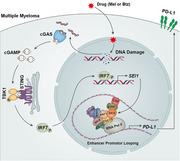

## 1 Introduction

Multiple myeloma (MM) is a malignancy that originates from malignant plasma cells in the bone marrow. This disease is predominantly found in the elderly, and its incidence has been escalating in recent years due to the growing aging population. As a result, it has become the second most common hematologic malignancy.^[^ ^1^ ^]^ Chemotherapy is the primary treatment for myeloma, and it commonly involves the use of several agents in clinical practice, including dexamethasone, doxorubicin, melphalan, prednisone, thalidomide, lenalidomide, and bortezomib.^[^ ^2^ ^]^ Although the current treatment has significantly improved, the disease remains incurable.^[^ ^2c^ ^]^ Research indicates that cancer treatment should not solely focus on eradicating cancer cells through radiotherapy and chemotherapy, but should also aim to enhance the patient's immune function. This includes inhibiting the function of immune checkpoint molecules and boosting the combat capability of T cells to strengthen the immune response against cancer.^[^ ^3^ ^]^ However, the understanding of how chemotherapy affects immune checkpoints and the immune microenvironment of myeloma cells is still limited. Therefore, there is an urgent need to investigate the relationship between chemotherapeutic drugs and immune evasion. Studies on the regulatory effects of chemotherapeutic drugs on immune checkpoints and the immune microenvironment of myeloma cells are essential for developing new, highly effective, and low‐side‐effect specific drugs. This study is critically important for improving both chemotherapy and immunotherapy for myeloma.

During immune editing, cancer cells evade immune surveillance by manipulating the immune checkpoint molecules. These molecules are essential for regulating immune activity, preventing autoimmunity and minimize tissue damage.^[^ ^4^ ^]^ Programmed death ligand‐1 (PD‐L1) plays a pivotal role in this process and is exploited by cancer cells to evade immune detection. When PD‐L1 on cancer cells and macrophages binds to programmed cell death protein‐1 (PD‐1) on activated cytotoxic T lymphocytes infiltrating tumors, it triggers an inhibitory signal that suppresses their antitumor activity.^[^ ^5^ ^]^ Furthermore, PD‐L1 is expressed at significantly higher levels in tumor tissues than in normal tissues. As a result, targeting the PD‐L1 and PD‐1 axis is viewed as a promising strategy for cancer immunotherapy.^[^ ^5^ ^]^ Therefore, comprehending the molecular mechanisms that regulating PD‐L1 expression is crucial for guiding the clinical application of PD‐1/PD‐L1 immune checkpoint inhibitors. Currently, research on the regulation of PD‐L1 in myeloma cells is relatively limited, with most studies primarily focusing on solid tumors and lymphomas. The primary regulatory mechanisms include chromatin and genomic alterations, transcriptional and post‐transcriptional regulation. Furthermore, various factors in the tumor microenvironment can induce PD‐L1 expression.

Chromatin modifications such as histone acetylation and methylation are important factors that cause changes in PD‐L1 expression. In breast cancer, TET2 protein recruits histone deacetylase 1 and 2 (HDAC1/2) to the PD‐L1 promoter, resulting in the deacetylation of histone H3 at lysine 27 (H3K27ac) and consequently repressing PD‐L1 transcription.^[^ ^6^ ^]^ Romidepsin, which targets HDAC1 and HDAC2, predominantly increases PD‐L1 expression in colorectal cancer through the modulation of histone H3 and H4 acetylation.^[^ ^7^ ^]^ In non‐small cell lung cancer, hepatocellular carcinoma, and breast cancer, a decrease in constitutive photomorphogenic 1 (COP1) levels leads to an accumulation of c‐Jun, which in turn suppresses the expression of histone deacetylase 3 (HDAC3), thereby enhancing histone H3 acetylation and stimulating PD‐L1 transcription.^[^ ^8^ ^]^ In liver cancer, enhancer of zeste homolog 2 (EZH2) downregulates PD‐L1 expression by directly increasing tri‐methylation of histone H3 at lysine 27 (H3K27me3) at the PD‐L1 and interferon regulatory factor 1 (IRF1) promoters, without impacting the activation of the interferon‐γ (IFNγ)–STAT1 signaling pathway.^[^ ^9^ ^]^ In liver cancer cells, lysine‐specific demethylase 1A (LSD1) interacts with MEF2D, reducing its methylation. The demethylated MEF2D then binds to the PD‐L1 promoter and activates its expression.^[^ ^10^ ^]^ Lu et al. discovered that histone methyltransferase mixed lineage leukemia 1 (MLL1) directly binds to the PD‐L1 promoter, catalyzing trimethylation of histone H3 lysine 4 (H3K4me3) and activating PD‐L1 transcription in tumor cells.^[^ ^11^ ^]^ MYC proto‐oncogene (MYC) is an oncogenic factor overexpressed in various tumors, and is involved in the regulation of PD‐L1 expression in multiple cancers.^[^ ^12^ ^]^

Post‐translational modifications significantly contribute to the regulation of PD‐L1. The primary mechanisms of such modifications in cells include ubiquitination,^[^ ^13^ ^]^ glycosylation,^[^ ^14^ ^]^ and phosphorylation,^[^ ^14^, ^15^ ^]^ which are collectively involved in the regulation of PD‐L1 expression. Beyond intrinsic tumor factors, the tumor microenvironment's excessive secretion of pro‐inflammatory cytokines, such as IFN‐γ,^[^ ^16^ ^]^ tumor necrosis factor α (TNF‐α),^[^ ^16^ ^]^ and interleukins (IL),^[^ ^17^ ^]^ can induce PD‐L1 expression in tumor cells, thereby facilitating immune escape. IFN‐γ primarily exerts its function in tumors through the Janus kinase (JAK)/STAT1/IRF pathway, while TNF‐α induces the expression of PD‐L1 mainly through the nuclear factor‐kappa B (NF‐κB) signaling pathway. In liver cancer cells, IL‐6 induces PD‐L1 expression via the JAK1 pathway.

Currently, there is a paucity of research on the regulation of PD‐L1 expression in myeloma. Existing studies suggested that the activation of the extracellular signal‐regulated kinase (ERK) pathway induces the expression of IFNγ, which subsequently stimulates the STAT1 pathway to promote PD‐L1 expression in myeloma cells.^[^ ^18^ ^]^ Moreover, it is posited that the JAK or p38–mitogen activated protein kinase (MAPK) pathway also promotes PD‐L1 expression in myeloma cells.^[^ ^19^ ^]^ The influence of chemotherapeutic drugs on alterations in PD‐L1 expression in myeloma cells, however, remains ambiguous. This ambiguity presents a significant concern regarding the interplay between chemotherapy and immune function in myeloma. Given these research gaps, there is an immediate requirement for more comprehensive investigations into the molecular biological mechanisms that govern the regulation of PD‐L1 expression in myeloma cells by chemotherapeutic drugs. Such an understanding is pivotal for the creation of new, effective, and low side‐effect specific drugs that can augment the efficacy of chemotherapeutic drugs and immune checkpoint inhibitors and circumvent drug resistance.

Through a combination of in vitro, in vivo, and patient's sample studies, we have reported that chemotherapy‐driven PD‐L1 upregulation promotes tumor immune evasion. After treatment of myeloma cells with chemotherapeutic drugs such as bortezomib or melphalan, DNA damage is induced, activating the cyclic guanosine monophosphate (GMP)‐adenosine monophosphate (AMP) synthase (cGAS)/stimulator of interferon genes (STING) signaling pathway. This activation leads to increased phosphorylation of the downstream transcription factor interferon regulatory factor 7 (IRF7), which binds to the promoter region of *SEI1* gene and promotes its transcription. The upregulated SEI1 interacts directly with the enhancer factors CREB‐binding protein (CBP)/p300 and RNA polymerase II (pol II)‐associated factor 1 (PAF1) complex, promoting PD‐L1 expression through transcription activation. This facilitates the immune escape of myeloma cells. Our findings not only provide insights into the mechanism of chemotherapy‐induced tumor immune evasion but also suggest a potential therapeutic strategy for myeloma patients by targeting SEI1.

## 2 Results

### 2.1 Chemotherapeutic Drugs Promote the Expression of PD‐L1 in Myeloma Cells and Inhibit Cytotoxic T‐Lymphocyte Activity

**Figure 1:**
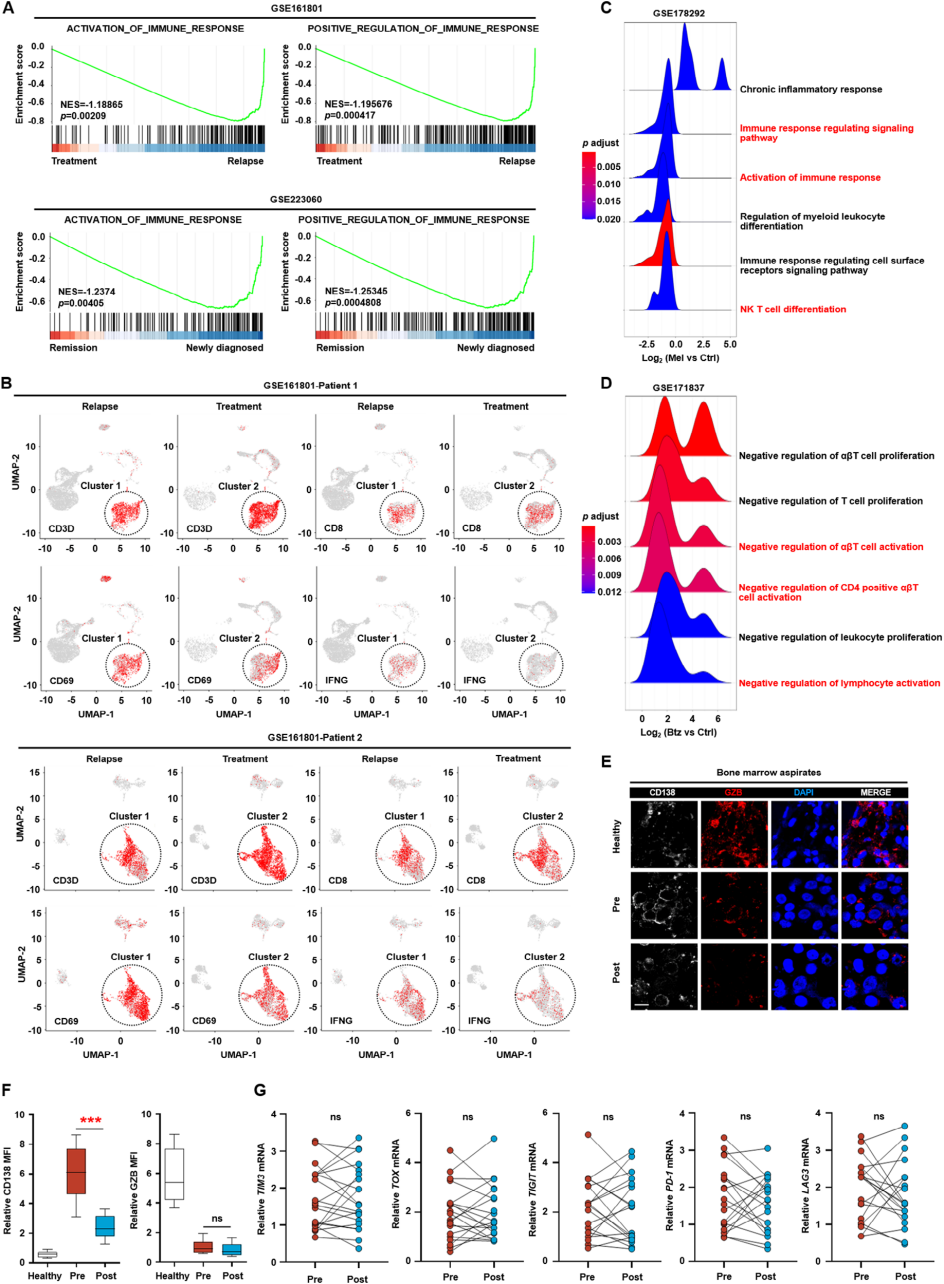
Chemotherapy inhibits cytotoxic T lymphocyte activity. A) Gene set enrichment analysis (GSEA) of public RNA‐seq datasets (GSE161801 (*n* = 30) and GSE223060 (*n* = 41)) of the immune‐response‐gene‐related signatures between newly diagnosed patients, undergoing treatment and those in relapse. NES, normalized enrichment score. B) Representative two patient's UMAP plot of the expression of *CD3D*, *CD8*, *CD69*, and *IFNG* in a public scRNA‐seq data (GSE161801) (*n* = 30). UMAP: uniform manifold approximation and projection. C,D) Pathway enrichment analysis of public RNA‐seq datasets of the myeloma cells treated with or without melphalan (GSE178292) or bortezomib (GSE171837). E) Immunofluorescent staining of healthy and myeloma patients bone marrow samples before or after chemotherapy with 4',6‐diamidino‐2‐phenylindole dihydrochloride (DAPI) and antibodies against CD138 (myeloma cell marker) or granzyme B (GZB, activity of T cell) (*n* = 5). Scale bar, 10 µm. F) Box plots showing the relative mean fluorescence intensity (MFI) of CD138 (left panel) or granzyme B (right panel) in the biopsy segment (*n* = 5). G) The relative expression of *TIM3*, *TOX*, *TIGIT*, *PD‐1*, or *LAG3* in CD8^+^ T cells isolated from the bone marrow aspirates of another 20 myeloma patients before or after chemotherapy.

Currently, chemotherapy is the primary treatment for myeloma. To elucidate the immunomodulatory effects of chemotherapy on the immune microenvironment in myeloma, we analyzed public RNA sequencing (RNA‐seq) datasets of the immune response genes between newly diagnosed patients, those undergoing treatment, and those in relapse patients. Using gene set enrichment analysis, we identified significant differences in the activation of immune response genes. Notably, the activation of these genes was enriched in newly diagnosed or relapsed patients compared to the treatment group, which suggests that chemotherapy significantly inhibits the activation of the immune system (**Figure** **1**A; Figure S1A,B, Supporting Information). Cytotoxic T lymphocytes (CTLs), particularly the CD8^+^ subset, are crucial components of the adaptive immune system, responsible for recognizing and eliminating infected or abnormal cells.^[^ ^20^ ^]^ Our next step was to confirm whether the chemotherapeutic drugs could affect the activation of CD8^+^ CTLs. To this end, we analyzed a public single‐cell RNA sequencing (scRNA‐seq) dataset of the heterogeneous tumor cell population of relapsed patients and its complex interaction network with the bone marrow microenvironment using scRNA‐seq after treatment. The results showed that chemotherapy significantly inhibited the activation of CD8^+^ CTLs (CD69^+^ and IFNγ^+^ population) (Figure 1B). Melphalan and bortezomib are commonly used clinical chemotherapeutic drugs for myeloma.^[^ ^2c^ ^]^ To further confirm our results, we analyzed public RNA‐seq datasets of myeloma cells treated with or without melphalan or bortezomib. The results also indicated that chemotherapy inhibited activation of T cells (Figure 1C,D; Figure S1C, Supporting Information). In addition, as the major effector of antitumor immunity, CD8^+^ CTLs eliminate cancer cells by secreting granzyme B (GZB).^[^ ^20^ ^]^ Thus, we examined CTL activity by measuring GZB release in bone marrow aspirates isolated from healthy donors and myeloma patients before or after treatment. We found that chemotherapy treatment reduced GZB release in the post‐treatment group, compared to the healthy group (Figure 1E,F). To further demonstrate the changes in the activity of CD8^+^ T cells before and after chemotherapy, we isolated bone marrow CD8^+^ T cells from additional 20 myeloma patients before and after chemotherapy. Quantitative polymerase chain reaction (qPCR) analysis was conducted to detect T‐cell exhaustion markers’ expressions (*TIM3*, *TOX*, *TIGIT*, *PD‐1*, and *LAG3*). The results indicated that CD8^+^ T cells showed a similar state of exhaustion, both exhibiting a low level of activity (Figure 1G).

**Figure 2:**
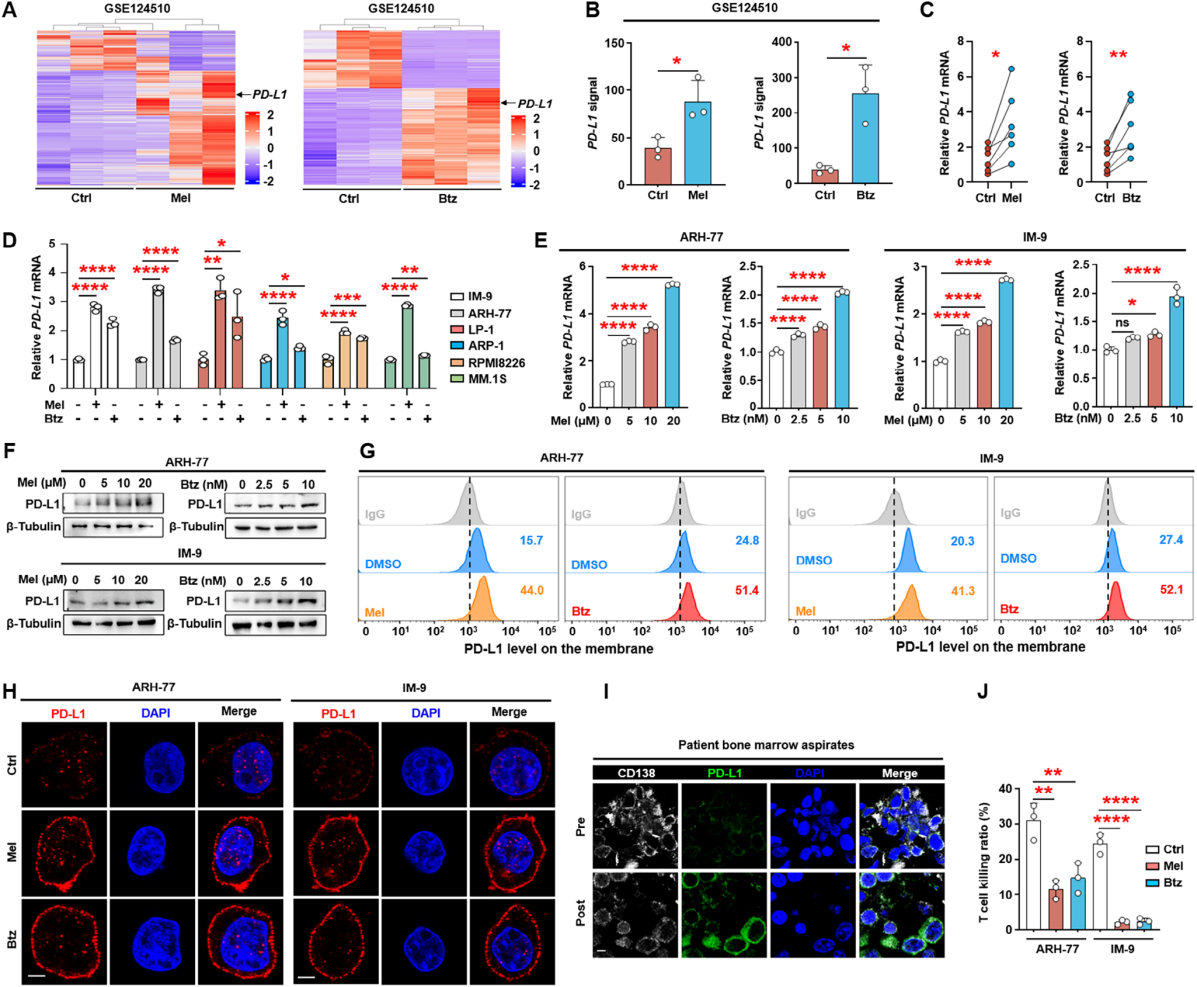
Melphalan or bortezomib promotes the expression of PD‐L1 in myeloma cells and inhibit cytotoxic T lymphocyte activity. A) A published GEO dataset (GSE124510) analysis shows the expression profile of upregulated genes in the MM.1S cells treated with or without melphalan or bortezomib. All gene names were listed in Table S7 (Supporting Information). B) *PD‐L1* messenger RNA (mRNA) levels of the MM.1S cells treated with or without melphalan or bortezomib (GSE124510). C,D) The expression of *PD‐L1* in primary myeloma cells (*n* = 6) or myeloma cell lines treated with or without melphalan or bortezomib. E) Relative mRNA and F) protein expression of PD‐L1 in ARH‐77 or IM‐9 cells treated with or without melphalan or bortezomib. G) Membrane PD‐L1 expression by flow cytometric analysis after melphalan (20 µm) or bortezomib (10 nm) treatment for 24 h. IgG, immunoglobulin G. H) Immunofluorescent staining of myeloma cell lines (ARH‐77 or IM‐9) with DAPI and antibodies against PD‐L1. Scale bar, 3 µm. I) Immunofluorescent staining of myeloma patients bone marrow samples before or after treatment with DAPI and antibodies against CD138 or PD‐L1 (*n* = 5). Scale bar, 5 µm. J) T‐cell‐mediated cancer cell killing assay. Pretreatment of myeloma cell lines with melphalan (20 µm) or bortezomib (10 nm) for 12 h, ARH‐77 or IM‐9 cells co‐cultured with activated T cell for 48 h were subjected to flow cytometric analysis. D–H,J) Representative of three independent experiments. Data are averages ± standard deviation (SD). *p < 0.05, **p < 0.01, ***p < 0.001, and ****p < 0.0001. B,C) *p*‐values were determined by Student's *t*‐test. D,E,J) *p*‐values were determined using one way ANOVA. ns, not significant.

Our next goal was to clarify how chemotherapeutic drugs affect the expression of immune checkpoint proteins in myeloma cells. We analyzed several public RNA sequencing datasets and found that melphalan or bortezomib promoted the expression of *PD‐L1* in myeloma cells, but did not change the expression of other common immune checkpoints (**Figure** **2**A,B; Figure S2A–C, Supporting Information). PD‐L1 is an important immune checkpoint molecule that is expressed on the surface of tumor cells. By binding to PD‐1, PD‐L1 can inhibit the function of T lymphocytes, thereby suppressing the self‐immune response. Blocking PD‐1/PD‐L1 can reactivate the cytotoxic T‐cell‐mediated killing of tumors, which is an important cancer immunotherapy approach.^[^ ^19^ ^]^ In addition, melphalan or bortezomib increased the expression of *PD‐L1* in primary myeloma cells and myeloma cell lines (Figure 2C,D). To further validate the association between melphalan or bortezomib and PD‐L1, we treated ARH‐77 or IM‐9 cells with increasing concentrations of melphalan or bortezomib. The expression of PD‐L1 was increased in a dose‐dependent manner (Figure 2E,F). Flow cytometry and immunofluorescence confirmed that the chemotherapeutic drugs significantly promote the expression of PD‐L1 on the membrane (Figure 2G,H). Moreover, prolonged treatment (96 h) with melphalan or bortezomib continued to upregulate the expression of PD‐L1 (Figure S3A–D, Supporting Information). Immunofluorescence analysis of bone marrow samples from myeloma patients before or after treatment indicated high expression of PD‐L1 in myeloma cells of post‐treatment group (Figure 2I). Pre‐treatment of myeloma cell lines with melphalan or bortezomib significantly inhibited T‐cell‐mediated cancer cell death, compared to the untreated group (Figure 2J). More importantly, public RNA‐seq dataset analysis indicated that other commonly used chemotherapeutic drugs in myeloma, such as carfilzomib or lenalidomide, can also promote the expression of *PD‐L1* (Figure S4A,B, Supporting Information). Collectively, these findings suggest that chemotherapeutic drugs increase PD‐L1 levels in cancer cells, which in turn diminish CTL activity against cancer cells.

### 2.2 Melphalan or Bortezomib Induces Upregulation of Myeloma Cell PD‐L1 Expression through Activation of DNA Damage and cGAS–STING Pathway

**Figure 3:**
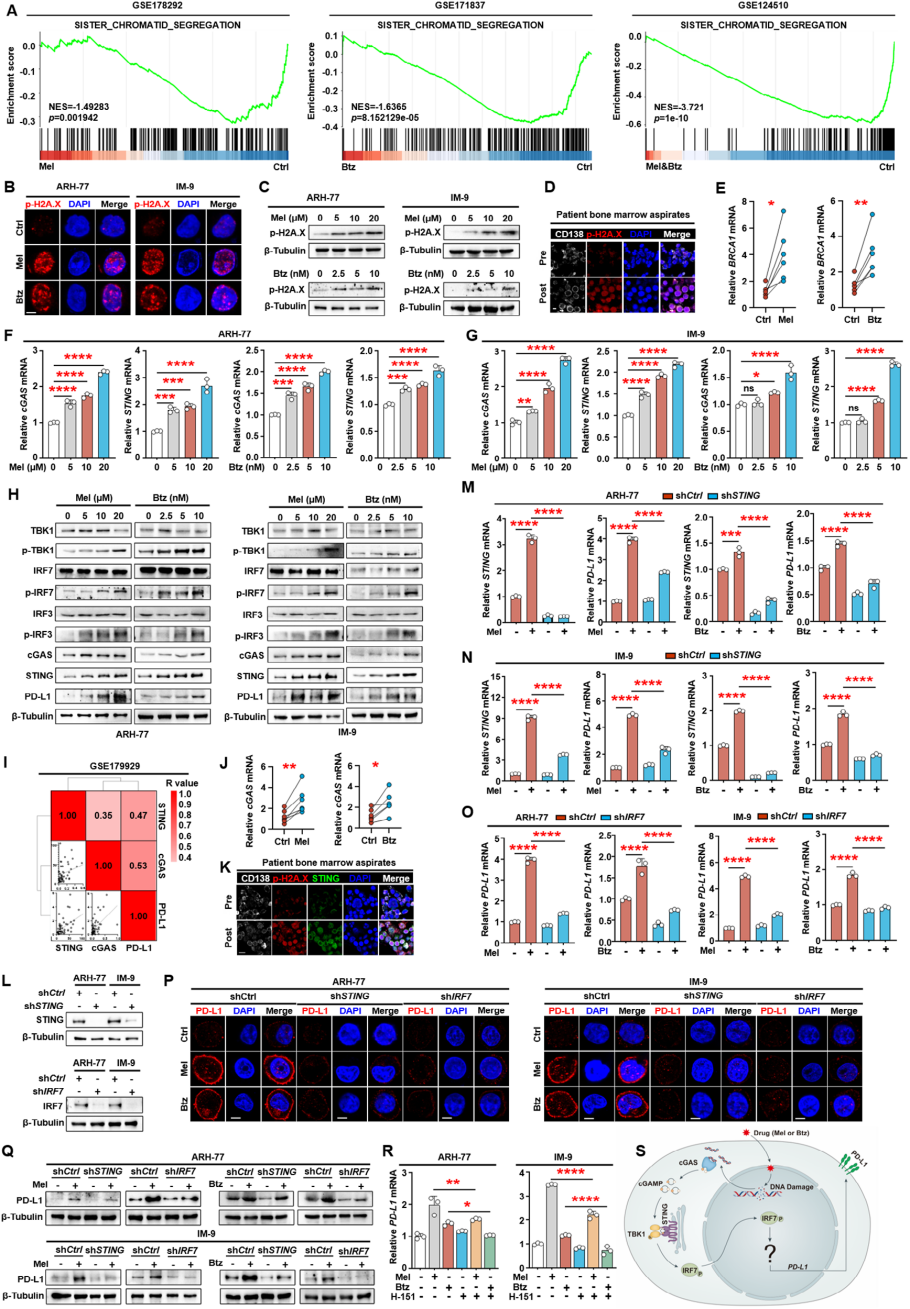
Melphalan or bortezomib induces upregulation of myeloma cell PD‐L1 expression through activation of DNA damage and cGAS–STING pathway. A) GSEA analysis of public RNA‐seq datasets (GSE178292, GSE171837, and GSE124510) of the sister chromatid segregation genes between myeloma cells treated with or without melphalan or bortezomib. B) Immunofluorescent staining of myeloma cell lines (ARH‐77 or IM‐9) with DAPI and antibodies against phosphorylated histone H2A.X. Scale bar, 3 µm. C) Western blot analysis of PD‐L1 in ARH‐77 or IM‐9 cells treated with or without melphalan or bortezomib. D) Immunofluorescent staining of myeloma patients’ bone marrow samples before or after treatment with DAPI and antibodies against CD138 or phosphorylated histone H2A.X. Scale bar, 3 µm. E) The relative expression of *BRCA1* in primary myeloma cells treated with or without melphalan or bortezomib (*n* = 6). F,G) The relative expression of *cGAS* or *STING* in ARH‐77 or IM‐9 cells treated with or without melphalan or bortezomib. H) Western blot analysis of cGAS–STING pathway‐associated proteins and PD‐L1 expression in ARH‐77 or IM‐9 cells treated with or without melphalan or bortezomib. I) Correlation coefficient of the mRNA levels of *PD‐L1* and the expression of *cGAS* or *STING* (*n* = 30) in a public RNA‐seq dataset (GSE179929). The correlations were evaluated using Pearson coefficient. *r*, correlation coefficient. J) The relative expression of *cGAS* in primary myeloma cells treated with or without melphalan or bortezomib (*n* = 6). K) Immunofluorescent staining of myeloma patients bone marrow samples before or after treatment with DAPI and antibodies against CD138, STING, or phosphorylated histone H2A.X (*n* = 5). Scale bar, 10 µm. L) Expression of STING or IRF7 in nonspecific, *STING* or *IRF7* shRNA‐expressing myeloma cells. M,N) The relative expression of *STING* or *PD‐L1* in STING knockdown ARH‐77 or IM‐9 cells treated with or without melphalan or bortezomib. O) The relative expression of *PD‐L1* in IRF7 knockdown ARH‐77 or IM‐9 cells treated with or without melphalan or bortezomib. P) Immunofluorescent analysis of ARH‐77 or IM‐9 myeloma cell lines with STING or IRF7 knockdown, treated with or without melphalan or bortezomib, and stained with DAPI and PD‐L1 antibody. Scale bar, 5 µm. Q) Western blot analysis of PD‐L1 in ARH‐77 or IM‐9 myeloma cell lines with STING or IRF7 knockdown, treated with or without melphalan or bortezomib. R) The relative expression of *PD‐L1* in ARH‐77 or IM‐9 myeloma cell lines, treated with or without melphalan, bortezomib, or H‐151. S) Schematic of the melphalan or bortezomib induces DNA damage/cGAS–STING/IRF7/PD‐L1 signaling axis. B,C,F–H,L–R) Representative of three independent experiments. Data are averages ± SD. *p < 0.05, **p < 0.01, ***p < 0.001, and ****p < 0.0001. E,J) *p*‐values were determined by Student's *t*‐test. F,G,M–O,R) *p*‐values were determined using one way ANOVA. ns, not significant.

Our next objective was to elucidate the mechanisms by which melphalan or bortezomib promotes PD‐L1 expression. First, we analyzed public RNA‐seq datasets to identify enrichment pathways in myeloma cells treated with or without melphalan or bortezomib using gene set enrichment analysis. We found significant differences in the genes involved in sister chromatid segregation. Notably, these genes were enriched in the untreated group, suggesting that chemotherapy significantly affected sister chromatid segregation (**Figure** **3**A). The proper segregation of chromosomes is essential for maintaining genome integrity during cell division. Failure to accurately untangle linked sister chromatids can result in the generation of bulky or ultrafine anaphase bridges, ultimately causing genome instability or damage.^[^ ^21^ ^]^ Immunofluorescence analysis of phosphorylated histone H2A.X levels in the nucleus indicated that the chemotherapeutic drugs induced DNA damage response (Figure 3B). To further confirm the correlation between melphalan or bortezomib and DNA damage, ARH‐77 or IM‐9 cells were exposed to escalating doses of melphalan or bortezomib. Results showed that both drugs increase phosphorylated histone H2A.X expression in a dose‐dependent manner (Figure 3C). Immunofluorescence analysis of bone marrow samples from myeloma patients before and after treatment also indicated high expression of phosphorylated histone H2A.X in myeloma cells after treatment (Figure 3D). Primary myeloma cells were isolated from the bone marrow aspirates of six patients treated with or without melphalan or bortezomib. qPCR analysis confirmed the upregulation of *BRCA1* (a DNA damage marker gene) in cells treated with drugs (Figure 3E). These data indicated that chemotherapeutic drug treatment induces DNA damage in myeloma cells.

cGAS is a cytosolic DNA sensor that elicits immune responses against microbial pathogens, such as DNA viruses.^[^ ^22^ ^]^ Upon activation, cGAS triggers the adapter protein STING, which subsequently recruits TRAF family member‐associated NF‐κB activator (TANK) binding kinase 1 (TBK1), IRF3, and IRF7, leading to the induction of type I IFN production. cGAS and STING are pivotal receptors for both exogenous and endogenous DNA.^[^ ^22^ ^]^ Subsequently, we investigated the potential of chemotherapy‐induced DNA damage in activating the cGAS–STING pathway. qPCR analysis demonstrated that a dose‐dependent increase in the expression of *cGAS* and *STING* in myeloma cells treated with melphalan or bortezomib (Figure 3F,G). Western blot results also demonstrated the activation of the cGAS–STING pathway by melphalan or bortezomib (Figure 3H). Furthermore, enzyme‐linked immunosorbent assay (ELISA) analysis demonstrated that melphalan or bortezomib enhanced the enzymatic activity of cGAS (Figure S5A,B, Supporting Information). In addition, analysis of public RNA‐seq datasets revealed a positive correlation between the expression of PD‐L1 and the expressions of *cGAS* and *STING* in primary myeloma cells (Figure 3I). qPCR analysis further confirmed the upregulation of *cGAS* in primary myeloma cells isolated from bone marrow aspirates of six myeloma patients treated with or without melphalan or bortezomib (Figure 3J). Immunofluorescence analysis of bone marrow samples from both pretreatment and post‐treatment myeloma patients demonstrated elevated expression of phosphorylated histone H2A.X and STING in myeloma cells of the post‐treatment group (Figure 3K). Further experimental validation confirmed that knocking down the expression of STING or IRF7 in myeloma cells significantly suppressed the expression of *STING* and *PD‐L1* induced by melphalan or bortezomib (Figure 3L–O). Similar results were obtained from western blotting and immunofluorescence experiments (Figure 3P,Q). The STING inhibitor also significantly attenuated the expression of *PD‐L1* induced by melphalan or bortezomib (Figure 3R). These findings demonstrate that melphalan or bortezomib upregulates PD‐L1 expression in myeloma cells by activating DNA damage and the cGAS–STING–IRF7 pathway (Figure 3S).

### 2.3 The cGAS–STING–IRF7 Signaling Axis Promotes SEI1 Expression, Which Upregulates PD‐L1 Expression

**Figure 4:**
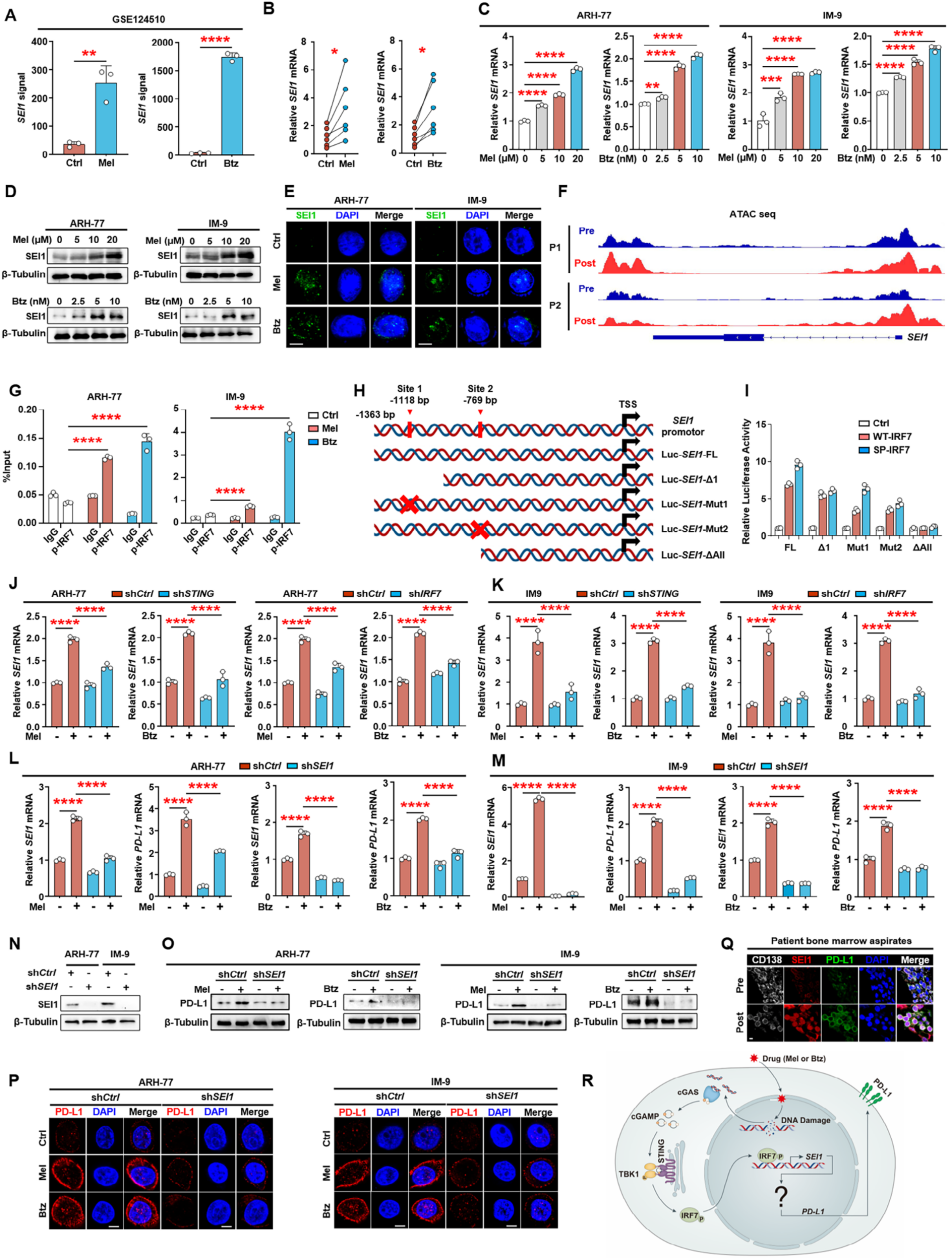
The cGAS–STING–IRF7 signaling axis promotes SEI1 expression, which upregulates PD‐L1 expression. A) A published GEO dataset (GSE124510) analysis shows *SEI1* mRNA levels of the MM.1S cells treated with or without melphalan or bortezomib. B) *SEI1* mRNA levels in primary myeloma cells treated with or without melphalan or bortezomib (*n* = 6). C) The relative expression of *SEI1* in ARH‐77 or IM‐9 cells treated with or without melphalan or bortezomib. D,E) Western blot and immunofluorescent analyses of SEI1 in ARH‐77 or IM‐9 cells treated with or without melphalan or bortezomib. Scale bar, 5 µm. F) Gene tracks showing representative ATAC‐seq profiles at *SEI1* gene loci in primary CD138^+^ plasma cells from two relapsed myeloma patients following treatment with bortezomib. G) ChIP PCR assay showing phosphor‐IRF7 enrichment on *SEI1* promoter of ARH‐77 or IM‐9 cells treated with or without melphalan or bortezomib. H,I) Schematic of the *SEI1* promoter luciferase reporter. Red crosses: mutations of nucleotides. The luciferase activity of Luc‐*SEI1* constructs was set at 1. SP‐IRF7, simulation of phosphorylation of IRF7. J,K) The relative expression of *SEI1* in STING or IRF7 knockdown ARH‐77 or IM‐9 cells treated with or without melphalan or bortezomib. L,M) The relative expression of *SEI1* or *PD‐L1* in SEI1 knockdown ARH‐77 or IM‐9 cells treated with or without melphalan or bortezomib. N–P) Western blot and immunofluorescent analysis of PD‐L1 in ARH‐77 or IM‐9 myeloma cell lines with SEI1 knockdown, treated with or without melphalan or bortezomib. Scale bar, 3 µm. Q) Immunofluorescent staining of myeloma patients bone marrow samples before or after treatment with DAPI and antibodies against CD138, SEI1, or PD‐L1 (*n* = 5). Scale bar, 5 µm. R) Schematic of the melphalan or bortezomib induces DNA damage/cGAS–STING/IRF7/SEI1/PD‐L1 signaling axis. C–E,G,I–P) Representative of three independent experiments. Data are averages ± SD. *p < 0.05, **p < 0.01, ***p < 0.001, and ****p < 0.0001. A,B) *p*‐values were determined by Student's *t*‐test. C,G,J,K) *p*‐values were determined using one way ANOVA. ns, not significant.

We next investigated the mechanism by which IRF7 increases PD‐L1 expression. Previous studies have reported that the downstream activation of the type I interferon or NF‐κB pathways by cGAS–STING can upregulate PD‐L1 expression.^[^ ^23^ ^]^ However, when myeloma cells were co‐treated with the NF‐κB inhibitor bortezomib and STAT pathway inhibitor nifuroxazide, the chemotherapeutic drugs still upregulated *PD‐L1* expression, indicating that IRF7 may have alternative regulatory pathways for upregulating PD‐L1 expression (Figure S6A,B, Supporting Information). To identify the molecules and pathways responsible for IRF7‐regulated PD‐L1 expression, we analyzed the Venn diagram of differentially expressed genes (DEGs) of the RNA‐seq data and found four genes upregulated in three public RNA‐seq datasets (*SEI1*, *CDKN1A*, *LMNA*, and *PDLIM2*) (Figure S7A,B, Supporting Information). SERTA domain‐containing protein 1 (SERTAD1), also known as transcriptional regulator interacting with plant homeodomain (PHD)‐bromodomain 1 (TRIP‐Br1) or SEI1, is a member of the SERTAD family and functions as an oncoprotein in many tumors.^[^ ^24^ ^]^ Other studies have reported an association between DNA damage and chromosomal instability.^[^ ^24^ ^]^ We aimed to determine whether the activation of IRF7 caused by chemotherapeutic drugs leads to the upregulation of PD‐L1 expression through SEI1. First, public RNA‐seq datasets’ analysis indicated that melphalan or bortezomib promoted the expression of *SEI1* mRNA (**Figure** **4**A). Upregulation of *SEI1* was also confirmed in primary myeloma cells isolated from bone marrow aspirates of six patients treated with or without melphalan or bortezomib (Figure 4B). qPCR analysis demonstrated that the expression of *SEI1* increased in a dose‐dependent manner in myeloma cell lines treated with melphalan or bortezomib (Figure 4C). Western blotting results also demonstrated increased expression of SEI1 induced by melphalan or bortezomib (Figure 4D). Additionally, similar results were obtained from immunofluorescence analysis (Figure 4E). The results demonstrate that melphalan or bortezomib promoted the expression of SEI1.

The next step was to demonstrate whether the activated IRF7–SEI1 axis can induce the expression of PD‐L1. Initially, we used an assay for transposase‐accessible chromatin with high‐throughput sequencing (ATAC‐seq) to examine the influence of chemotherapeutic drugs on chromatin accessibility in myeloma cells. Specifically, we assessed the chromatin accessibility of primary CD138^+^ plasma cells from two relapsed myeloma patients following treatment with bortezomib, and confirmed a significant enhancement in the intensity of the ATAC‐seq signal at the transcription start site (TSS) regions of the *SEI1* gene (Figure 4F). Promoter sequence analysis indicated that there are two putative binding sites for IRF7 on the *SEI1* promoter. Therefore, we investigated whether phosphorylated IRF7 could regulate *SEI1* transcription. Chromatin‐immunoprecipitation (ChIP) PCR analysis showed that melphalan or bortezomib treatment increased the enrichment of phosphorated IRF7 on *SEI1* promoter (Figure 4G), indicating that phosphorylated IRF7 induced *SEI1* transcription. To demonstrate how phosphorylated IRF7 binds to the *SEI1* promoter, we examined a 1.3 kilobase region around the *SEI1* gene transcriptional start site. Based on the promoter sequence, we predicted two IRF7 binding site (−1118 base pairs (bp) and −769 bp) on the *SEI1* gene promoter (Figure 4H). We generated two truncated forms of the *SEI1* gene promoter. Deletion mapping showed that phosphorylated IRF7 binds to both sites. Mutating both putative motifs confirmed that these loci were IRF7‐binding sites (Figure 4I). Subsequent experimental validation corroborated that downregulating the expression of STING or IRF7 in myeloma cells markedly attenuates the induction of *SEI1* by melphalan or bortezomib (Figure 4J,K), and downregulation of *SEI1* reduced the induction of *PD‐L1* by melphalan or bortezomib (Figure 4L,M). Furthermore, consistent findings were obtained from western blotting and immunofluorescence experiments (Figure 4N–P). Moreover, immunofluorescence analysis of bone marrow samples from both pretreatment and post‐treatment myeloma patients demonstrated elevated expression of SEI1 and PD‐L1 in the myeloma cells of the post‐treatment group (Figure 4Q). Through co‐culture experiments with activated T cells, we found that overexpression of SEI1 in myeloma cells can significantly inhibit the killing efficiency of activated T cells (Figure S8A–D, Supporting Information) and promote the expression of T‐cell exhaustion markers (*TOX*, *TIGIT*, and *TIM3*) (Figure S8E,F, Supporting Information). Furthermore, ChIP PCR analysis also indicated that melphalan or bortezomib promotes SEI1 transcription without involving the phosphorylation of p65, IRF3, or histone methylation modifications (Figure S9A–F, Supporting Information). Together, these results show that the cGAS–STING–IRF7 signaling axis facilitates the expression of SEI1, leading to the upregulation of PD‐L1 expression (Figure 4R).

### 2.4 SEI1 Bridges the Interaction between CBP/p300 and PAF1 Complex to Regulate PD‐L1 Transcription

**Figure 5:**
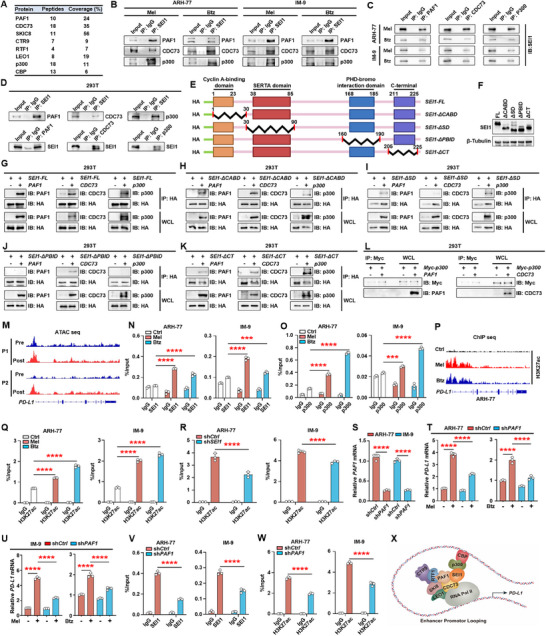
SEI1 bridges the interaction between CBP/p300 and PAF1 complex to regulate PD‐L1 transcription. A) *HA‐SEI1* overexpressed IM‐9 cells were lysed, and the total protein lysate was immunoprecipitated with an agarose‐immobilized HA antibody and analysis with mass spectrometry. The proteins identified are indicated. B,C) Co‐immunoprecipitation of SEI1, PAF1, CDC73, or p300 in ARH‐77 or IM‐9 cells treated with melphalan or bortezomib. D) Co‐immunoprecipitation of SEI1 along with PAF1, CDC73, or p300 in HEK293T cells co‐transfected with *SEI1* and either *PAF1*, *CDC73*, or *p300* plasmid. E) Schematic of the truncations including ΔCABD, ΔSD, ΔPBID, and ΔCT fragments. F) Western blotting showing different truncations of SEI1 (full length, ΔCABD, ΔSD, ΔPBID, and ΔCT) in HEK293T cells. G–K) Pulldown of PAF1, CDC73, or p300 with different truncations of SEI1 (full length, ΔCABD, ΔSD, ΔPBID, and ΔCT) in HEK293T cells. L) Pulldown of MYC‐p300 with CDC73 or PAF1 in HEK293T cells. M) Gene tracks showing representative ATAC‐seq profiles at *PD‐L1* gene loci in primary CD138^+^ plasma cells from two relapsed myeloma patients following treatment with bortezomib. N,O) ChIP PCR assay showing SEI1, p300, or H3K27ac enrichment on *PD‐L1* promoter of ARH‐77 or IM‐9 cells treated with or without melphalan or bortezomib. P) ChIP‐seq profiles show H3K27ac signal tracks at the *PD‐L1* gene loci for ARH‐77 cells treated with or without melphalan or bortezomib. Q) ChIP PCR assay showing H3K27ac enrichment on *PD‐L1* promoter of ARH‐77 or IM‐9 cells treated with or without melphalan or bortezomib. R) ChIP PCR assay showing enrichment of H3K27ac on *PD‐L1* promoter in sh*Ctrl* or sh*SEI1* ARH‐77 or IM‐9 cells. S) Expression of *PAF1* in nonspecific, *PAF1* shRNA‐expressing myeloma cells. T,U) The relative expression of *PD‐L1* in PAF1 knockdown ARH‐77 and IM‐9 cells treated with or without melphalan or bortezomib. V,W) ChIP PCR assay showing enrichment of V) SEI1 or W) H3K27ac on *PD‐L1* promoter in sh*Ctrl* or sh*PAF1* ARH‐77 and IM‐9 cells. X) Schematic of the SEI1 bridges the interaction between CBP/p300 and PAF1 complex to regulate PD‐L1 transcription. B–D,F–L,N,O,Q–W) Representative of three independent experiments. Data are averages ± SD. ***p < 0.001 and ****p < 0.0001. All *p*‐values were determined using one way ANOVA.

In order to elucidate the molecular mechanism by which SEI1 regulates PD‐L1, we performed immunoprecipitation and mass spectrometry analyses. These analyses identified CBP/p300 and PAF1 complex (PAF1, CDC73, SKIC8, CTR9, RTF1, and LEO1) proteins as specific binding partners of SEI1 (**Figure** **5**A; Table S1, Supporting Information). CBP/p300 catalyzes the dynamic activation of superenhancers via histone acetylation.^[^ ^25^ ^]^ Moreover, the PAF1 complex is a highly conserved transcription regulatory complex that interacts with RNA polymerase II and participates in multiple stages of transcription regulation, such as transcriptional pausing and elongation.^[^ ^26^ ^]^ Previous studies have reported that the PAF1 complex regulates promoter‐proximal pause release via enhancer activation.^[^ ^26^ ^]^ We propose that SEI1 promotes the transcription of PD‐L1 by interacting with superenhancer factors and the PAF1 complex. To investigate the presence of the endogenous SEI1/CBP/p300/PAF1 complex in myeloma cells, we performed immunoprecipitation assays using anti‐SEI1, PAF1, CDC73, or p300 antibodies on myeloma cell lysates. We examined the lysates for the presence of PAF1, CDC73, or p300. Our results confirmed the presence of PAF1, CDC73, or p300 in the immunoprecipitates, indicating the existence of the SEI1/CBP/p300/PAF1 complex in myeloma cells. Additionally, by immunoprecipitation using anti‐PAF1, CDC73, or p300 antibodies, we detected the presence of SEI1 in the immunocomplexes (Figure 5B,C). To further examine these interactions, we performed co‐immunoprecipitation assays. HEK293T cells were co‐transfected with plasmids expressing SEI1 along with PAF1, CDC73, or p300. Immunoprecipitation was performed, and the immunoprecipitates were analyzed for the presence of PAF1, CDC73, or p300 proteins. Similarly, when HEK293T cells were co‐transfected with plasmids expressing PAF1, CDC73, or p300 along with SEI1, we also found SEI1 protein in the immunoprecipitates (Figure 5D). The results demonstrate that SEI1 interacted with PAF1, CDC73, and p300.

Previous studies have identified four relatively conserved domains within the primary sequence of the SEI1 protein that are responsible for protein–protein interactions: the cyclin A‐binding domain (CABD), SERTA domain (SD), PHD‐bromo interaction domain (PBID), and C‐terminal domain (CT).^[^ ^27^ ^]^ To determine which domain of SEI1 interacts with PAF1, CDC73, or p300, we selectively knocked out these four domains (ΔCABD, ΔSD, ΔPBID, and ΔCT) (Figure 5E,F). Pulldown assays showed that SEI1 interacts with CDC73 through the SERTA domain, and interacts with PAF1 or p300 through the PHD‐bromo interaction domain (Figure 5G–K). Additionally, the pulldown assay demonstrated no direct interactions between p300 and the PAF1 or CDC73 proteins (Figure 5L). Furthermore, ATAC‐seq analysis of two relapsed myeloma patients following treatment with bortezomib showed that enhancement in the intensity of signal at the TSS regions of *PD‐L1* gene (Figure 5M). ChIP PCR analysis showed that increased enrichment of SEI1 or p300 on the *PD‐L1* gene promoter following treatment with melphalan or bortezomib (Figure 5N,O). We queried the H3K27ac status with genome‐wide chromatin immunoprecipitation sequencing (ChIP‐seq). We found enrichment of H3K27ac on the *PD‐L1* gene promoter in myeloma cells treated with melphalan or bortezomib (Figure 5P). ChIP PCR analysis further confirmed that melphalan or bortezomib increased enrichment of H3K27ac on *PD‐L1* gene promoter (Figure 5Q). Knocking down SEI1 expression reduced enrichment of H3K27ac on *PD‐L1* gene promoter (Figure 5R). Further experiments confirmed that knocking down the expression of PAF1 in myeloma cells significantly suppressed the expression of *PD‐L1* induced by melphalan or bortezomib (Figure 5S–U). Knocking down PAF1 expression also reduced enrichment of SEI1 or H3K27ac on *PD‐L1* promoter (Figure 5V,W). Additionally, ATAC‐seq of myeloma cell lines following treatment with lenalidomide also showed that enhancement in the intensity of signal at the TSS regions of *PD‐L1* gene (Figure S10A,B, Supporting Information). ChIP‐seq analysis showed enrichment of H3K27ac on the *PD‐L1* gene promoter in myeloma cells treated with lenalidomide (Figure S10C, Supporting Information). Further ChIP PCR analysis revealed that phosphorylated NF‐κB p65 activated by melphalan or bortezomib can also promote PD‐L1 transcription, which is consistent with the findings in Figures S6 and S11A (Supporting Information). However, neither of them promotes PD‐L1 transcription through the phosphorylation of IRF3 or histone methylation (Figure S11B–E, Supporting Information). These results showed that SEI1 facilitates the interaction between CBP/p300 and the PAF1 complex to modulate *PD‐L1* transcription (Figure 5X).

### 2.5 SEI1 Facilitates Myeloma Immune Evasion through Chemotherapy‐Induced PD‐L1 Induction, Both In Vivo and In Clinical Relevance

**Figure 6:**
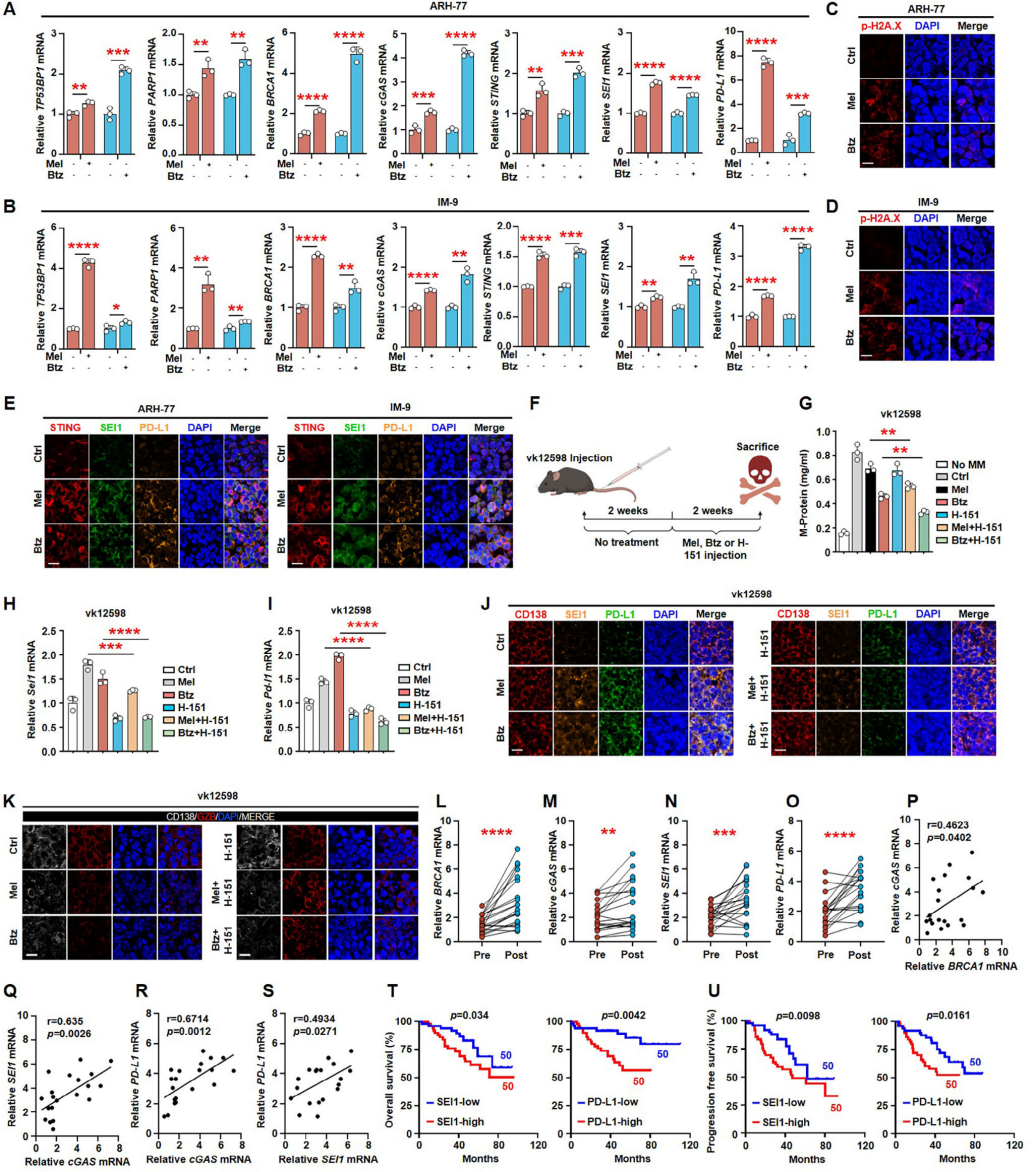
SEI1 facilitates tumor immune evasion through chemotherapy‐induced PD‐L1 induction, both in vivo and in clinical relevance. A,B) ARH‐77 or IM‐9 cell lines were subcutaneously injected into the NSG mice, and intraperitoneal administration of bortezomib (0.5 mg kg^−1^ bodyweight) or melphalan (1 mg kg^−1^ bodyweight) three times per week for a duration of 2 weeks, beginning 2 weeks after cell injection. Shown are the relative expressions of *TP53BP1*, *PARP1*, *BRCA1*, *cGAS*, *STING*, *SEI1*, and *PD‐L1* in tumor tissue of NSG mice (*n* = 3 mice per group). C,D) Immunofluorescent staining of a mentioned subcutaneous tumor with DAPI and antibodies against phosphorylated histone H2A.X. Scale bar, 10 µm. E) Immunofluorescent staining of a mentioned subcutaneous tumor with DAPI and antibodies against STING, SEI1, or PD‐L1. Scale bar, 10 µm. 6 week old male C57BL/6J mice were intravenously injected vk12598 cells (*n* = 3 mice per group), followed by intraperitoneal administration of bortezomib (0.5 mg kg^−1^ bodyweight), melphalan (1 mg kg^−1^ bodyweight), or H‐151 (10 mg kg^−1^ bodyweight) three times per week for a duration of 2 weeks, beginning 2 weeks after cell injection. F) The experimental schematic. G) ELISA analysis shown the concentrations of M‐protein in mouse sera. H,I) The relative expressions of *SEI1* or *PD‐L1* in spleen tumor cells of vk12598 mice (*n* = 3 mice per group). J) Immunofluorescent staining of F mentioned spleen tumor cells of vk12598 mice with DAPI and antibodies against CD138, SEI1, or PD‐L1. Scale bar, 10 µm. K) Immunofluorescent staining of F mentioned spleen tumor cells of vk12598 mice with DAPI and antibodies against CD138 or granzyme B. Scale bar, 10 µm. L–O) The relative expression of *BRCA1*, *cGAS*, *SEI1*, or *PD‐L1* in primary myeloma cells isolated from the bone marrow aspirates of 20 myeloma patients before or after chemotherapy. P–S) Correlation coefficient of the mRNA levels of *cGAS* and mRNA levels of *BRCA1*, *SEI1*, or *PD‐L1* or mRNA levels of *SEI1* and mRNA levels of *PD‐L1* in myeloma patients (*n* = 20). The correlations were evaluated using Pearson coefficient. *r*, correlation coefficient. *p*‐value was determined by Pearson coefficient. T,U) Kaplan–Meier analysis of T) overall and U) progression‐free survivals in myeloma patients with high (*n* = 50) or low (*n* = 50) *SEI1* or *PD‐L1* expression. Data are averages ± SD. *p < 0.05, **p < 0.01, ***p < 0.001, and ****p < 0.0001. M–P) *p*‐values were determined by Student's *t*‐test. A,B,G–I) *p*‐values were determined using one way ANOVA. T,U) *p‐*values were determined using a two‐sided log‐rank test.

To further extend our findings to an in vivo study, we injected ARH‐77 or IM‐9 cell lines subcutaneously into immunodeficient NOD Scid gamma (NSG) mice, and administered chemotherapeutic drugs intraperitoneally three times per week for 2 weeks, starting 2 weeks after cell injection. qPCR analysis of the tumor tissue demonstrated that the expressions of DNA damage marker genes (*TP53BP1*, *PARP1*, and *BRCA1*), *cGAS*, *STING*, *SEI1*, and *PD‐L1* were increased in melphalan or bortezomib treated mice (**Figure** **6**A,B). Immunofluorescence analysis of tumor tissues showed similar results (Figure 6C–E). In order to further observe the impact of chemotherapy drugs on immunocompetent myeloma mouse models, we also intravenously injected vk*MYC cell line (vk12598) cells into C57BL/6J mice, followed by intraperitoneal administration of chemotherapeutic drugs or STING inhibitor (H‐151) three times per week for a duration of 2 weeks, beginning 2 weeks after cell injection. Mice that did not receive myeloma cells served as controls (Figure 6F). After 4 weeks, we measured the establishment of myeloma by detecting increased levels of circulating M‐proteins, an indicator of myeloma burden. We observed that the combination of chemotherapeutic drugs with STING inhibitor further reduced tumor burden (Figure 6G) and *SEI1* or *PD‐L1* expression in myeloma cells (Figure 6H,I) compared with the use of chemotherapeutic drugs alone. Immunofluorescence analysis also showed that STING inhibitor treatment reduced melphalan‐ or bortezomib‐enhanced SEI1 or PD‐L1 expression (Figure 6J), and increased GZB release (Figure 6K). All these results confirmed that SEI1 facilitates tumor immune evasion through chemotherapy‐induced PD‐L1 induction. Finally, to assess the clinical relevance of chemotherapy and PD‐L1 expression in myeloma patients, qPCR analysis confirmed the upregulation of *BRCA1*, *cGAS*, *SEI1*, and *PD‐L1* expressions in primary myeloma cells isolated from the bone marrow aspirates of 20 myeloma patients before and after treatment (Figure 6L–O). We further analyzed primary myeloma cells from 20 treated patients and found a positive correlation between the *cGAS* expression and *BRCA1*, *SEI1*, or *PD‐L1* expression (Figure 6P–R). A head‐to‐head comparison of *SEI1* and *PD‐L1* expressions demonstrated a positive correlation (Figure 6S). More importantly, patients with high expression of SEI1 or PD‐L1 exhibited poor overall and progression‐free survival (Figure 6T,U). More importantly, the clinical data analysis of the patients in Figure 6T showed that compared to myeloma patients with low SEI1 expression, those with high SEI1 expression have faster disease progression, higher levels of lactate dehydrogenase in their serum, and a lower proportion of achieving complete remission after initial treatment (Table S2, Supporting Information).

To further expand the clinical relevance of our findings, we aim to investigate whether our conclusions are applicable to other tumors treated with melphalan or bortezomib. qPCR analysis confirmed the upregulation of *BRCA1*, *cGAS*, *STING*, *SEI1*, and *PD‐L1* expressions in the breast cancer cell lines (Figure S12A,B, Supporting Information) or lymphoma cell lines (Figure S12C–F, Supporting Information) treated with melphalan or bortezomib. These results demonstrate that similar molecular mechanisms of chemotherapy‐induced immune escape are prevalent in both breast cancer and lymphoma.

### 2.6 Sequential Treatment with Chemotherapy Followed by PD‐L1 Antibodies on Myeloma Cells’ Augment‐Activated T‐Cell Cytotoxicity

**Figure 7:**
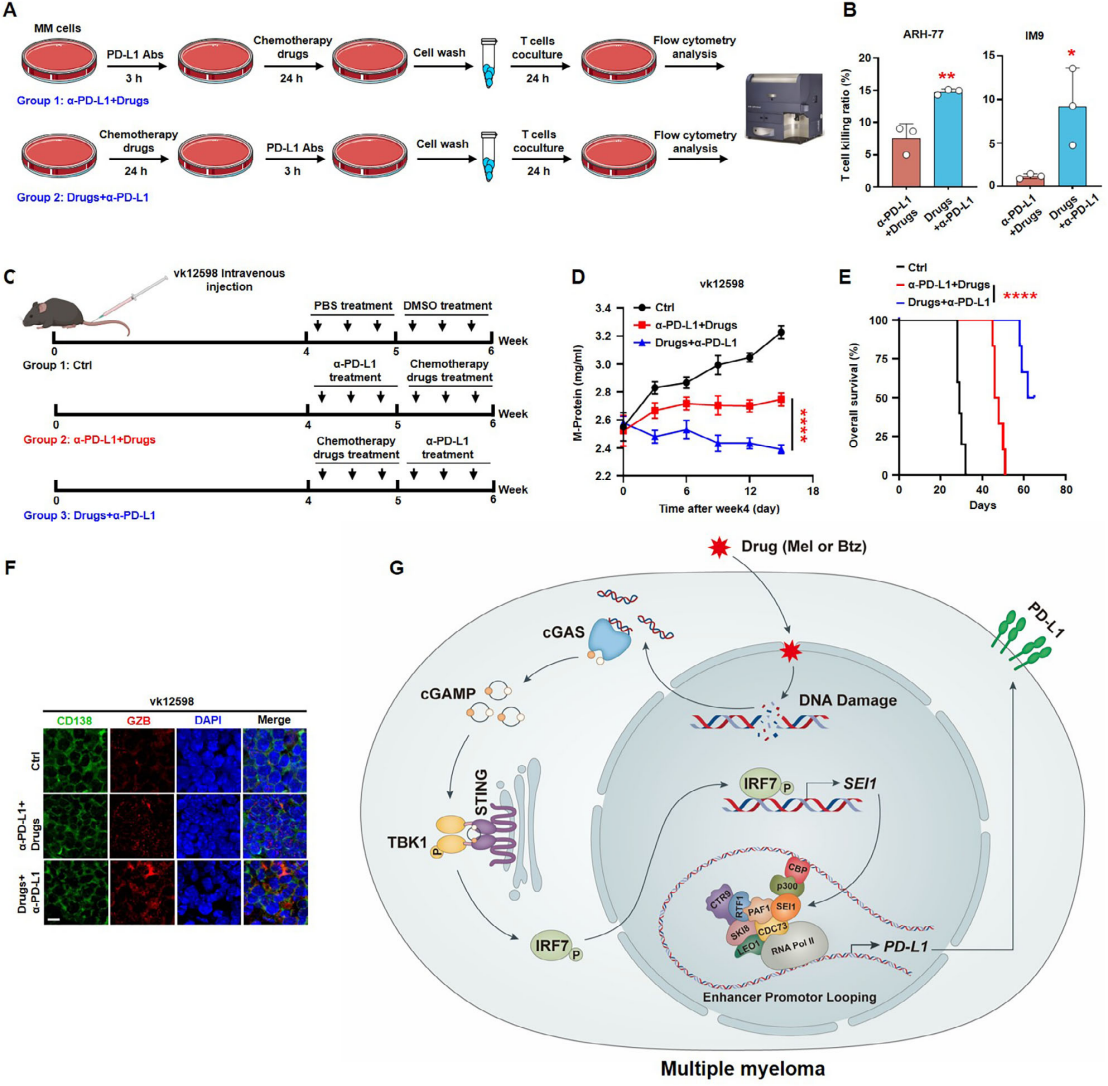
Sequential treatment with chemotherapy followed by PD‐L1 antibodies on myeloma cells augments activated T cells cytotoxicity. A,B) T‐cell‐mediated cancer cell killing assay. Myeloma cells (ARH‐77 or IM‐9) were pretreated with melphalan (10 µm) and bortezomib (5 nm) or PD‐L1 antibodies (1 µg mL^−1^) in various sequences, followed by co‐culture with activated T cells for 24 h were subjected to flow cytometric analysis (*n* = 3 biological replicates). C) 6 week old male C57BL/6J mice were intravenously injected vk12598 cells (*n* = 3 mice per group), followed by intraperitoneal administration of bortezomib (0.5 mg kg^−1^ bodyweight) and melphalan (0.5 mg kg^−1^ bodyweight) or PD‐L1 antibodies (10 mg kg^−1^ bodyweight) in different sequences three times for 1 week each. D) ELISA analysis shown the concentrations of M‐protein in mouse sera. E) Kaplan–Meier analysis of overall mouse survival (*n* = 5 mice per group). F) Immunofluorescent staining of mouse spleen samples using DAPI, CD138 antibodies, or granzyme B (GZB) antibodies. Scale bar, 5 µm. G) Depiction of signaling pathways involved in SEI1 facilitates tumor immune evasion through chemotherapy‐induced PD‐L1 induction. Data are averages ± SD. *p < 0.05, **p < 0.01, and ****p < 0.0001. B) *p*‐value was determined by the Student's *t*‐test. D) *p*‐value was determined using two‐way ANOVA. E) *p‐*value was determined using a two‐sided log‐rank test.

Based on our findings, we explored the optimal combination of chemotherapy and PD‐L1 antibody therapy. We first performed an in vitro T‐cell killing assay, pretreating myeloma cells with chemotherapy, or PD‐L1 antibodies in different sequences, followed by co‐culture with activated T cells (**Figure** **7**A). Apoptosis analysis demonstrated that treating myeloma cells with PD‐L1 antibodies after chemotherapy significantly enhances the cytotoxic efficiency of activated T cells compared to the reverse sequence (Figure 7B). In vivo, we intravenously injected vk12598 cells into C57BL/6J mice and, and after 4 weeks, treated them with chemotherapy drugs or PD‐L1 antibodies in different sequences for 1 week each (Figure 7C). The results indicated that administering chemotherapy prior to PD‐L1 antibody treatment significantly inhibited the progression of myeloma and prolonged the survival time compared to the reverse sequence (Figure 7D,E). Additionally, we assessed CTL activity by measuring GZB release in spleen cells isolated from treated mice. The results showed that administering chemotherapy prior to PD‐L1 antibody treatment increased GZB release, compared to the reverse sequence (Figure 7F). Furthermore, the combination therapy proved significantly more effective than chemotherapy or PD‐L1 antibody monotherapy (Figure S13A–D, Supporting Information). These findings suggest that the upregulation of PD‐L1 expression induced by chemotherapy enhances the sensitivity of myeloma cells to PD‐L1 antibody therapy, which has important implications for the clinical application of PD‐L1 antibodies.

## 3 Discussion

In contrast to cytotoxic T lymphocyte antigen 4 (CTLA‐4) or PD‐1, which is predominantly expressed in immune cells, PD‐L1 is expressed in cancer cells and macrophages, playing a crucial role in suppressing immune surveillance.^[^ ^19^ ^]^ This study elucidates the mechanisms by which chemotherapeutic drugs induce PD‐L1 expression, leading to the initiation of T‐cell immunosuppression by cancer cells. We found that chemotherapeutic drugs such as melphalan and bortezomib cause DNA damage in myeloma cells, which activates the cGAS/STING signaling pathway. This activation increases the phosphorylation level of the downstream transcription factor IRF7, which then binds to the promoter region of *SEI1* gene to promote its transcription. The elevated expression of SEI1 directly interacts with the CBP/p300 and PAF1 transcriptional regulatory complexes to promote PD‐L1 expression, resulting in immune evasion in myeloma cells (Figure 7G).

Currently, researches on the cGAS–STING pathway and its downstream regulation of PD‐L1 have yielded significant insights. For instance, TBK1 and TBK binding protein 1 (TBKBP1) have been shown to regulate tumor‐mediated immunosuppression by facilitating epidermal growth factor (EGF)‐induced PD‐L1 expression.^[^ ^28^ ^]^ Additionally, the STING–TBK1 axis triggers an innate immune response by phosphorylating IRF3, IRF7, or NF‐κB. It has been reported that NF‐κB can directly induce PD‐L1 transcription by binding to its promoter and regulate PD‐L1 post‐transcriptionally through indirect pathways.^[^ ^29^ ^]^ Furthermore, the NF‐κB/IRF3 complex has been found to be enriched on the PD‐L1 promoter and is responsible for the transcriptional upregulation of PD‐L1.^[^ ^23^ ^]^ Moreover, IRF3‐activated type I interferon also activates the STAT pathway to promote PD‐L1 transcription.^[^ ^30^ ^]^ However, it is currently unclear whether other signaling pathways downstream of the cGAS–STING pathway are involved in the regulation of PD‐L1 expression. Our study found that the combined use of an NF‐κB inhibitor bortezomib and a STAT inhibitor nifuroxazide could not completely inhibit the upregulation of PD‐L1 induced by chemotherapeutic drugs, indicating the involvement of other molecular mechanisms. Our research suggests that the phosphorylation of IRF7 enhances PD‐L1 transcription by regulating SEI1. Our study expands the understanding of how cGAS–STING signaling pathway regulates PD‐L1 expression.

As a critical determinant of transcription elongation, the PAF1 complex has been implicated in multiple facets of RNA polymerase II‐mediated transcription, including initiation, pausing, elongation, and termination.^[^ ^26^ ^]^ Depletion of the PAF1 complex enhances the release of RNA polymerase II, resulting in the upregulation of some genes, while also blocking transcriptional elongation, leading to downregulation of gene expression. This effect is more pronounced for longer genes, where the decrease in expression levels was more significant.^[^ ^31^ ^]^ Therefore, the PAF1 complex regulates gene expression by controlling transcriptional pausing and elongation. Additionally, other studies have also demonstrated that a subset of enhancers primarily regulates gene expression by controlling the release of paused RNA polymerase II in a PAF1‐dependent manner.^[^ ^26^ ^]^ They observed PAF1 complex occupancy not only at active promoters characterized by high levels of H3K4me3, but also at active enhancers marked by H3K27ac and H3 Lys4 mono‐methylation (H3K4me1).^[^ ^26^ ^]^ In our study, we found that SEI1 interacted with both PAF1 complex and CBP/p300, and the expression level of SEI1 also influenced the enrichment of H3K27ac on the PD‐L1 promoter. Therefore, the interaction between SEI1 and these proteins plays a crucial role in PAF1 complex‐mediated enhancer transcriptional activity, which is important for understanding the relationship between enhancer transcriptional regulation and PAF1 complex. This work further enriches our understanding of the regulatory mechanisms of the PAF1 complex.

SEI1 is a member of the TRIP‐Br family, functions as a nuclear factor that regulates the cell cycle by interacting with cyclin‐dependent kinase 4 (CDK4) and E2F/DP‐1. As an oncogene, SEI1 is associated with mechanisms that lead to chromosomal instability, such as increasing chromosomes numbers, double minute chromosomes, and micronuclei formation.^[^ ^24^ ^]^ Furthermore, SEI1 inhibits apoptosis and promotes metastasis through PI3K‐Akt or integrin‐linked kinase (ILK) modulation in HER2/neu‐positive and neu‐negative cancer cells.^[^ ^32^ ^]^ However, the role of SEI1 in myeloma remains unclear. Our work revealed that SEI1 acts as a bridge between enhancers and the PAF1 complex, exerting transcriptional regulation to promote the expression of PD‐L1. This provides valuable insights into improving the therapeutic efficacy of chemotherapy in myeloma and has also uncovered a new regulatory mechanism for PD‐L1 in myeloma cells. Additionally, previous studies have reported that the efficacy of PD‐L1 blockade in myeloma patients is limited.^[^ ^33^ ^]^ One possible reason is that some myeloma patients have low expression levels of PD‐L1 on their tumor cells, rendering them insensitive to PD‐L1 antibody therapy. According to our research, combining PD‐L1 antibody therapy with chemotherapy or administering PD‐L1 antibodies after chemotherapy is likely to enhance the therapeutic effect of PD‐L1 antibodies. Our findings hold positive implications for the application of PD‐L1 antibodies in myeloma patients and for improvements in myeloma treatment.

PD‐L1 plays a pivotal role in the immune microenvironment, particularly within the tumor microenvironment (TME), where it modulates interactions with various immune cells. The upregulation of PD‐L1 on tumor cells and immune cells within the TME has significant implications for immune evasion and the overall antitumor immune response.^[^ ^5^ ^]^ PD‐L1 interacts with PD‐1 on immune cells, leading to the suppression of T‐cell activation, proliferation, and cytokine secretion, which facilitates the immune escape of tumor cells.^[^ ^5^ ^]^ In addition to tumor cells, PD‐L1 is expressed on immune cells such as T cells, B cells, and natural killer (NK) cells, as well as on myeloid cells including macrophages, myeloid‐derived suppressor cells, and dendritic cells (DCs) within the TME.^[^ ^34^ ^]^ Upregulation of PD‐L1 on these cells also contributes to the suppression of antitumor T‐cell responses and is critical for stratifying patients for their response to anti‐PD‐1/PD‐L1 immunotherapy. Tumor‐associated macrophages (TAMs) secrete cytokines such as IFN‐γ and TNF, which upregulate PD‐L1 expression on tumor cells.^[^ ^16^ ^]^ The interaction of PD‐1 and PD‐L1 negatively regulates adaptive immune responses mainly by inhibiting the activity of effector T cells while enhancing the function of regulatory T cells (Tregs).^[^ ^16^ ^]^ This can lead to an immunesuppressive phenotype in macrophages, which is often associated with poor prognosis.^[^ ^16^ ^]^ DCs also express high levels of PD‐L1 in the TME, and while the physiological functions of PD‐L1 on DCs remain incompletely understood, it is known that PD‐L1 can protect DCs from being killed by PD‐1‐expressing T cells, thereby potentially impairing the antigen‐presenting function of DCs.^[^ ^35^ ^]^ In summary, PD‐L1 upregulation in the TME has a complex impact on the interactions between tumor cells and various immune cells, including T cells, macrophages, and DCs. This upregulation contributes to the immunosuppressive nature of the TME and can influence the efficacy of immunotherapies targeting the PD‐1/PD‐L1 axis.^[^ ^36^ ^]^ Our work discovered that chemotherapy upregulates the expression of PD‐L1 in myeloma cells, but it is still unclear whether it can regulate the PD‐L1 expression on T cells, macrophages, DCs, B cells, or NK cells. In the future, we plan to study the regulatory effects of chemotherapy on PD‐L1 or other immune checkpoints in various immune cells, to further deepen our understanding of the regulatory role of chemotherapy on the immune microenvironment, and to provide more treatment options for myeloma chemotherapy and immunotherapy.

Despite the significant insights gained from this study, there are noteworthy limitations that should be acknowledged. First, our research primarily relies on the mining of public sequencing data, which lacks new original sequencing data, thereby limiting the novelty and robustness of our study. For instance, in single‐cell sequencing analysis, we lack detailed knowledge about patient backgrounds and specific treatment regimens. The complexity of treatment protocols and the drugs used may introduce biases into our analysis. Therefore, in our subsequent research plan, we will collect a sufficient number of bone marrow aspirates from patients for single‐cell analysis and meticulously document patient information and treatment regimens. Additionally, the number of clinical samples used in this study is limited, raising concerns about the generalizability of the proposed mechanisms. Although we included an additional analysis of the exhaustion status of CD8^+^ T cells isolated from 20 patients and examined the correlation between SEI1 and overall survival and progression‐free survival of myeloma patients, our study still requires further validation in a larger sample size. In subsequent work, we will increase the sample size and engage in more extensive collaborative research to better understand the relationship between chemotherapy and myeloma immune escape. Furthermore, although durvalumab (an anti‐PD‐L1 antibody) is currently being evaluated in combination with lenalidomide in clinical trials involving patients with newly diagnosed myeloma (NCT02685826),^[^ ^33^ ^]^ additional long‐term followup data are not yet available to assess the impact of combined chemotherapy or immunomodulatory agents with PD‐L1 antibodies on long‐term prognostic indicators. In future work, it will be essential to validate the efficacy of this combination therapy in a larger cohort of patients. Finally, given that the tumor microenvironment is a complex ecosystem comprising various cell types, including macrophages, fibroblasts, endothelial cells, and extracellular matrix components, all of which play critical roles in tumor progression and therapy response, our current study primarily focuses on the molecular pathways within cancer cells. However, the interactions between cancer cells and other tumor microenvironment components, particularly during chemotherapy‐induced immune escape, are of great importance. For instance, TAMs and cancer‐associated fibroblasts (CAFs) are known to secrete cytokines and growth factors that can modulate PD‐L1 expression on cancer cells and contribute to the establishment of an immunosuppressive microenvironment.^[^ ^33^, ^37^ ^]^ Whether chemotherapy drugs influence the regulatory roles of TAMs and CAFs in shaping the immunosuppressive microenvironment of myeloma is a compelling question that deserves in‐depth exploration in future studies.

## 4 Experimental Section

### Cell Lines, Primary Myeloma Cells, and CD8^+^ T Cells

Myeloma cell lines (ARH‐77 and IM‐9) and lymphoma cell lines (JeKo‐1 and Jurkat) were provided by Dr. Zhiqiang Liu's lab at Shandong First Medical University and Shandong Academy of Medical Sciences. MCF‐7, MDA‐MB‐231, and HEK293T cells were purchased from the American Type Culture Collection (ATCC). The murine myeloma vk12598 was provided by the Mayo Clinic. Primary myeloma cells were isolated from the bone marrow aspirates of myeloma patients by using anti‐CD138 antibody‐coated magnetic beads (Miltenyi Biotec). CD8^+^ T cells were isolated from the bone marrow aspirates of myeloma patients by using CD8^+^ T Cell Isolation Kit (Miltenyi Biotec). Informed written consent from all participants was obtained. Myeloma and lymphoma cells were maintained in RPMI1640 medium with 10% fetal bovine serum (FBS). MCF‐7, MDA‐MB‐231, and HEK293T cells were cultured in Dulbecco's modified Eagle's medium (DMEM) with 10% FBS. Patient samples were obtained from the First Affiliated Hospital of Xiamen University, China. This study was approved by the Ethics Committee of Xiamen University (XMULAC20210081), and all protocols conformed to the Ethical Guidelines of the World Medical Association Declaration of Helsinki.

### Antibodies, Plasmids, and Reagents

The plasmids *SEI1*, *p300*, *CDC73*, *PAF1*, and control vector were purchased from Genocopies. Human *SEI1‐ΔCABD*, *SEI1‐ΔSD*, *SEI1‐ΔPBID*, or *SEI1‐ΔCT* was subcloned into a pLVx vector. Except where specified, all chemicals were purchased from Sigma–Aldrich, and antibodies for western blot analysis were purchased from Cell Signaling Technology. STING inhibitor H‐151 was purchased from TargetMol, USA (T5674). Short hairpin RNAs (shRNAs) targeting primers against *STING*, *SEI1*, *IRF7*, and nontarget control were purchased from Sigma–Aldrich. sh*SEI1*, sh*IRF7*, and sh*STING* were subcloned into a pLKO.1 vector. The primers used for the amino acid deletion fragment of SEI1 and shRNAs targeting primers are listed in Table S3 (Supporting Information).

### Stable Cell Line Construction

Transient transfections of HEK 293T cells were conducted using polyethyleneimine (PEI) (Polysciences, Warrington) in OPTI‐MEM medium (Life Technologies) at a 1:4 DNA‐to‐PEI ratio. Viral particles were generated in HEK 293T cells co‐transfected with 4 µg of pMD2.G (#12259) and 6 µg of psPAX2 (#12260) packaging plasmids sourced from Addgene, along with 8 µg of lentiviral vectors expressing target genes, including human *SEI1* and pLKO.1 vector for shRNAs targeting *SRING*, *IRF7*, *PAF1*, or *SEI‐1*. The viral supernatant was harvested 48 h following transfection and subjected to a 100‐fold concentration using polyethylene glycol 8000 from Sigma–Aldrich. Myeloma cells were transduced via spinfection with the concentrated viral particles in conjunction with polybrene, at a centrifugal force of 800 × *g* for 30 min at a temperature of 37 °C. After 12 h post infection, the medium was refreshed, and the cells were further cultured for an additional 48 h prior to subsequent processing. The establishment of stable cell lines was achieved by incorporating puromycin (2 µg mL^−1^) (#540222, Sigma–Aldrich) into the culture medium.

### Western Blot Analysis

Cells were harvested and lysed with 1 × lysis buffer (#9803, Cell Signaling Technology). Cell lysates were subjected to sodium dodecyl sulfate‐polyacrylamide gel electrophoresis (SDS‐PAGE), transferred to a nitrocellulose membrane, and immunoblotted with antibodies against β‐tubulin (#2128), PD‐L1 (#13684), phosphorylated histone H2A.X (#9718), phosphorylated TBK1 (#5483), TBK1 (#3504), phosphorylated IRF7 (#12390), IRF7 (#39659), phosphorylated IRF3 (#29047), IRF3 (#11904), cGAS (#79978), STING (#13647), PAF1(#12883), CDC73(#8126), p300 (#86377), MYC‐Tag (#2278), and HA‐Tag (#3724) (Cell Signaling Technology). SEI1 antibody was purchased from Santa Cruz (sc‐517080).

### qPCR of mRNAs

Total RNA was isolated using an RNeasy kit (QIAGEN). An aliquot of 1 µg total RNA was subjected to reverse transcription (RT) using a SuperScript II RT‐PCR kit (Invitrogen). qPCR was performed using SYBR Green Master Mix (Life Technologies) and the QuantStudio 3 Real‐Time PCR System (Life Technologies). The reaction was performed using the following settings: 95 °C for 10 min, followed by 40 cycles of 95 °C for 15 s and 60 °C for 60 s. Glyceraldehyde‐3‐phosphate dehydrogenase (GAPDH) served as endogenous controls. The primers used are listed in Table S4 (Supporting Information).

### Flow Cytometry and ELISA

Mouse bone marrow cells were fixed and stained with PD‐L1 antibody (BD Biosciences, #568079), and measured using a BD LSRFortessa flow cytometer. The results were analyzed using Flow Jo software. ELISA kits were purchased from R&D system, Cayman Chemical and Immunodiagnostic Systems.

### T‐Cell‐Mediated Tumor Cell Killing Assay

To acquire activated T cells, human peripheral blood mononuclear cells (PBMCs) were cultured in RPMI1640 complete medium with human CD3/CD28 antibodies (R&D System) and IL‐2 (10 ng mL^−1^) (R&D System) for 1 week. The ratios between cancer cells and activated cells were 1:5. After co‐culture, T cells and cancer cells were collected, and apoptosis of cancer cells was detected by annexin V–fluorescein isothiocyanate (FITC)/propidium iodide (PI) staining according to the manufacturer's instructions (eBioscience).

### Immunoprecipitation and Pulldown Assays

Cells were lysed and incubated on ice for 15 min. The total protein lysate was immunoprecipitated with an agarose‐immobilized antibody at 4 °C overnight. After washing six times, the beads were resuspended in 30 µL of 1 × SDS buffer. After boiling for 5 min, pulldown samples were run on SDS‐PAGE gel along with a 5% input sample and transferred to a polyvinylidene fluoride (PVDF) membrane for immunoblotting. Immunoglobulin G (IgG) was used as a control, and total cell lysates were used as input controls. For the pulldown assay, HEK293T cells were transfected with either HA‐tag protein plasmid. Lysates of the cells pulled down with HA beads were further incubated with cell lysates transfected with *CDC73*, *PAF1*, or *p300* plasmid. The immunoprecipitates and whole cell lysates (WCL) were immunoblotted. Cells that were not transfected with plasmid or WCL served as controls.

### ChIP, ChIP‐Seq, and RNA‐Seq

Cells were fixed in 4% formaldehyde and sonicated to prepare chromatin fragments. Chromatin samples were immunoprecipitated with antibodies against phosphorylated IRF7 (#12390), acetyl‐histone H3 (Lys27) (H3k27ac) (#8173), p300 (#54062), phosphorylated p65 (#3033), trimethyl‐histone H3 (Lys4) (H3K4me3) (#9751), trimethyl‐histone H3 (Lys27) (H3k27me3) (#9733), trimethyl‐histone H3 (Lys36) (H3k36me3) (#4909), phosphorylated IRF3 (#29 047) (Cell Signaling Technology), SEI1 (sc‐517080, Santa Cruz), and control IgG at 4 °C for 3 h. Immunoprecipitates and total chromatin inputs were reverse cross‐linked; DNA was isolated and analyzed using PCR with primers. The primer sequences used are listed in Table S5 (Supporting Information).

About 20 ng of ChIP DNA immunoprecipitated with anti‐H3K27ac antibodies or control IgG from parallel experiments was used for library preparation and sequencing using Illumina kit (#IP‐102‐1001). The libraries were then aliquoted onto a HiSeq 2000 platform at a concentration of 6 pm and underwent 50 cycles of sequencing. Quality assessment and initial data processing of raw reads from both ChIP and input libraries were conducted using FastQC (version 0.11.8) and Cutadapt (version 2.0) to derive high‐quality reads. Subsequently, these reads were aligned to the *Homo sapiens* reference genome (hg38) using Bowtie2 (version 2.2.6). SAMtools (version 1.9) was subsequently applied to eliminate duplicate reads. The identification of regions exhibiting substantial ChIP signal enrichment was accomplished through the peak‐finding algorithm of MACS2 (version 2.1.1). Parameters for read mapping and peak calling were calibrated according to the Encyclopedia of DNA Elements (ENCODE) Processing Pipeline for histone modification ChIP‐Seq. To correlate the identified peaks with proximal genes and genomic regions, the annotatePeaks.pl script from HOMER (version 4.10) was implemented.

RNA‐seq raw data were retrieved from the Gene Expression Omnibus (GEO) repository. The integrity of the raw reads was initially assessed employing FastQC (version 0.11.8). Subsequently, adapter removal and quality trimming were performed using Cutadapt (version 2.0) to yield cleaned datasets. For gene expression analysis, the *Homo sapiens* genome sequence and gene annotation data (hg38, GRCh38) were sourced from the GENCODE project. Quantification of gene expression was conducted using featureCounts (version 1.6.0). Differential gene expression analysis between sample cohorts was executed with DESeq2 (utilizing *R* version 3.3.2). The resulting DEGs were refined by applying a stringent threshold: a minimum fold change of 1 and a maximum adjusted *p*‐value of 0.05.

### Fluorescent Staining

Myeloma cells were fixed with 4% formaldehyde and permeabilized with 0.3% Triton X‐100 in 1 × phosphate buffered saline (PBS). After blocking with 2% goat serum, the cells were incubated with anti‐PD‐L1 (#13684, Cell Signaling Technology), phosphorylated histone H2A.X (#9718, Cell Signaling Technology), or SEI1 (#sc‐517080, Santa Cruz) antibodies overnight at 4 °C; cells were incubated with FITC‐conjugated goat antimouse IgG (H+L) (#AS001, ABclonal) or Cy3‐conjugated goat antirabbit IgG (H+L) (#AS007, ABclonal) for 60 min at room temperature and nucleus counterstaining with DAPI. Formalin‐fixed, paraffin‐embedded sections of bone marrow biopsy samples obtained from myeloma patients or subcutaneous tumors in myeloma mice were deparaffinized and stained. Slides were stained with quadruple‐fluorescence immunohistochemical mouse/rabbit kit (ImmunoWay) following the manufacturer's instructions. CD138 antibody was purchased from R&D Systems (#AF3190). Granzyme B antibody was purchased from Cell Signaling Technology (#17215). Immunofluorescent images were acquired with an IX71 confocal microscope system (Olympus). Mean fluorescence intensity (MFI) was quantified using ImageJ software (v 2.1.0/1.53c). The average fluorescence intensity within each region for each subject was derived by calculating the quotient of the cumulative fluorescence intensity across all observed fields to the aggregate area of those fields within the specified region. For each distinct signal, the average fluorescence intensity was ascertained per slide, with a minimum of six distinct areas per tumor being analyzed.

### Mass Spectrometry

Hemagglutinin (HA) tagged SEI1 protein (HA‐SEI1) overexpressed IM‐9 cells were lysed and incubated on ice for 15 min. The total protein lysate was immunoprecipitated with an agarose‐immobilized HA antibody at 4 °C overnight. After washing six times, the elution protein samples were analyzed using a nano‐liquid chromatography/tandem mass spectrometry (LC/MS/MS) (Thermo Fisher Scientific) coupled with an 1100 high‐performance liquid chromatography (HPLC) (Agilent Technologies). The MS/MS spectra were searched using the SEQUEST software program with the BioWorks Browser (version 3.3.1; Thermo Fisher Scientific) against the NCBI database. IM‐9 cells transfected with an empty vector served as controls. The mass spectrometry proteomics data were deposited to the ProteomeXchange Consortium via the PRIDE partner repository with the dataset identifier PXD052783.

### Luciferase Assay In Vitro

The construct covers the full‐length (Luc‐*SEI1*), two truncated forms (Luc‐*SEI1*‐Δ1 and Luc‐*SEI1*‐Δ2), and two mutated forms (Luc‐*SEI1*‐mut1 and Luc‐*SEI1*‐mut2) made around the promoter of the *SEI1* gene were subcloned into the pGL2 vector, and their transcriptional activities in HEK293T cells were examined using a dual‐luciferase reporter assay system (Promega) according to the manufacturer's instructions. The luciferase activity of Luc‐*SEI1* constructs was set at 1. The primers used in the subcloning and simulation of phosphorylation of IRF7 are listed in Table S6 (Supporting Information).

### In Vivo Mouse Experiments

NSG mice and C57BL/6J mice were purchased from Shanghai Model Organisms Center, Shanghai, China, were maintained in Xiamen University Animal Care‐accredited facilities. The mouse studies were approved by the Institutional Animal Care and Use Committee of Xiamen University. Myeloma cells (ARH‐77 or IM‐9) (1 × 10^6^ cells per mouse) were intravenously injected into NSG mice, and intraperitoneal administration of bortezomib (0.5 mg kg^−1^ bodyweight) or melphalan (1 mg kg^−1^ bodyweight) three times per week for a duration of 2 weeks, beginning 2 weeks after cell injection. The tumor tissues were collected and subjected to qPCR or immunofluorescence analysis. 6 week old female C57BL/6J mice were intravenously injected vk12598 cells, followed by intraperitoneal administration of chemotherapeutic drugs bortezomib (0.5 mg kg^−1^ bodyweight), melphalan (1 mg kg^−1^ bodyweight), or H‐151 (10 mg kg^−1^ bodyweight) three times per week for a duration of 2 weeks, beginning 2 weeks after cell injection. Mice that did not receive myeloma cells served as controls. After 4 weeks, the establishment of myeloma was measured by detecting increased levels of circulating M‐proteins. The spleen was collected and subjected to qPCR or immunofluorescence analysis.

vk12598 cells were intravenously injected into C57BL/6J mice, and after 4 weeks, treated them with chemotherapy drugs or PD‐L1 antibodies in different sequences for 1 week each. Mice injected with PBS or dimethyl sulfoxide (DMSO) served as controls. After 6 weeks, the establishment of myeloma was measured by detecting M‐proteins. The spleen was collected and subjected to immunofluorescence analysis.

### Statistical Analysis

Statistical significance was assessed using Graphpad software (Version 9.0). Unpaired Student's *t*‐tests were employed for comparing two groups, while one‐way analysis of variance (ANOVA) with Tukey's multiple comparisons test was used for comparing more than two groups. Kaplan–Meier analysis was used in survival analysis.

Unpaired Student's *t*‐tests and *χ* ^2^‐square tests were used to analyze the characteristics of myeloma patients. A *p*‐value of less than 0.05 was considered indicative of statistically significant differences. All findings were verified using a minimum of three independent experiments.

## Conflict of Interest

The authors have no competing interests.

## Author Contributions

R.C. and Z.L. contributed equally to this work. H.L., R.L., and Z.Q.L. designed all experiments and wrote the manuscript. R.C., Z.W.L., R.L., Z.H.F., Z.L., D.Y.Y., Y.L., and S.R.L. performed experiments and statistical analysis. Z.H.F. provided patient samples. All authors reviewed the final manuscript.

## Supporting information



Supporting Information

Supporting Information

## Data Availability

The data that support the findings of this study are available from the corresponding author upon reasonable request. The ChIP‐seq data generated in this study are available at GEO database (PRJNA1110794).
