# Supplementary material for: Chemotherapy‐Mediated Induction of PD‐L1 via SEI1 Facilitates Myeloma Immune Evasion
Source: Adv Sci (Weinh). 2025 Mar 26;12(19):2411082. doi: 10.1002/advs.202411082 (PMC12097018; doi:10.1002/advs.202411082)
Supplement: Supplementary file 2 — Supporting Information [file ADVS-12-2411082-s001.docx]

**Supplementary Figures and Figure Legends**


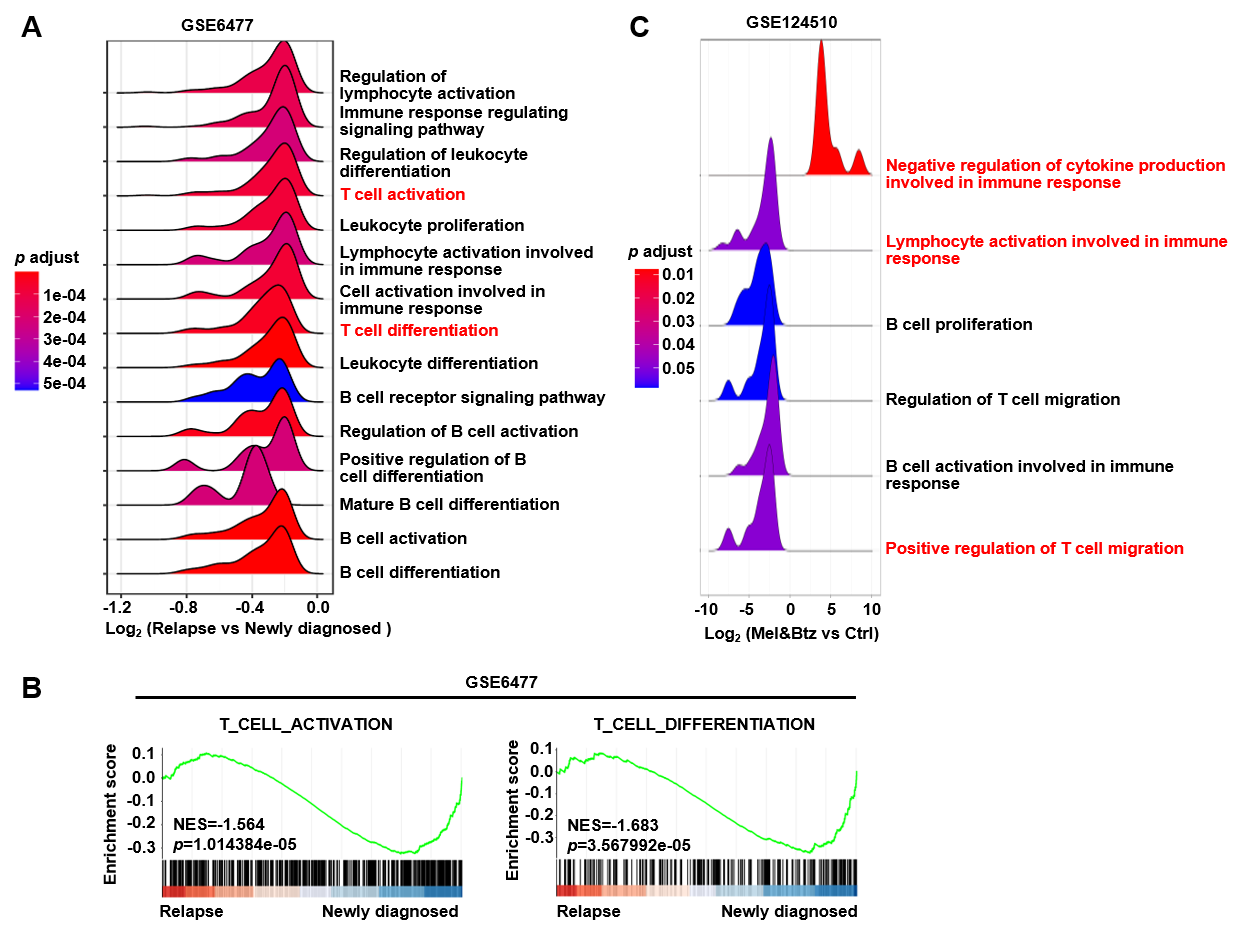


**Figure S1. Chemotherapy hinders the function of cytotoxic T lymphocytes.**

(**A**) Pathway enrichment analysis of public RNA-seq datasets of the patient myeloma cells isolated from newly diagnosed or relapsed patients (GSE6477) (Newly diagnosed, n = 73; Relapse, n = 29). (**B**) GSEA analysis of public RNA-seq datasets (GSE6477) of the T cell activation and T cell differentiation between newly diagnosed patients and those in relapse. (**C**) Pathway enrichment analysis of public RNA-seq dataset of the myeloma cells treated with or without melphalan and bortezomib (GSE124510) (n = 3 biological replicates).


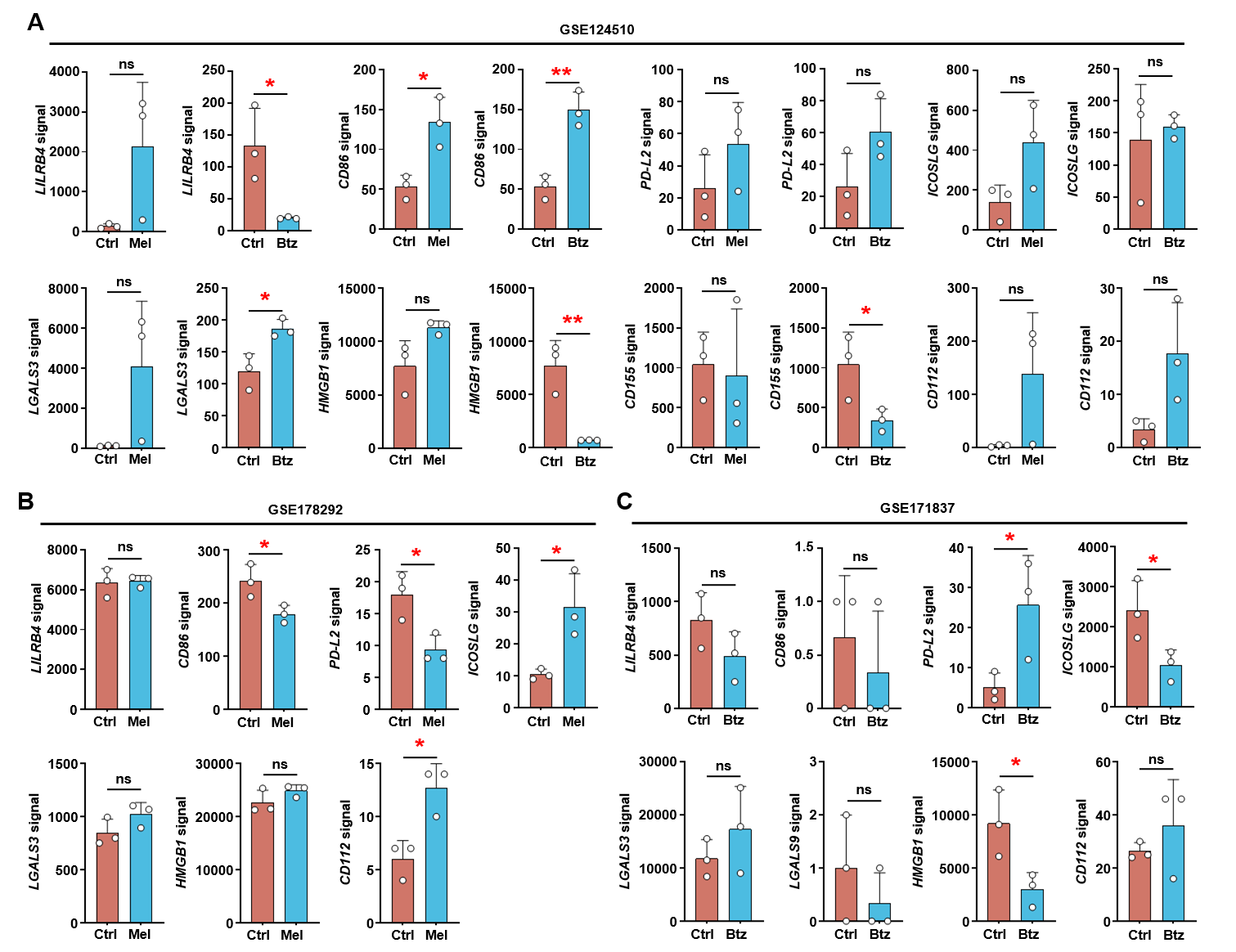


**Figure S2. The expression of other immune checkpoint proteins in myeloma cells after treatment with melphalan or bortezomib.**

(**A**) *LILRB4*, *CD86*, *PD-L2*, *ICOSLG*, *LGALS3*, *HMGB1*, *CD155* and *CD112* mRNA levels of the MM.1S cells treated with or without melphalan or bortezomib (n = 3 biological replicates). (**B**) *LILRB4*, *CD86*, *PD-L2*, *ICOSLG*, *LGALS3*, *HMGB1* and *CD112* mRNA levels of the INA-6 cells treated with or without melphalan (GSE178292) (n = 3 biological replicates). (**C**) *LILRB4*, *CD86*, *PD-L2*, *ICOSLG* *LGALS3*, *LGALS9*, *HMGB1* and *CD112* mRNA levels of the AMO1 cells treated with or without bortezomib (GSE171837) (n = 3 biological replicates). **p* < 0.05, ***p* < 0.01. All *p* values were determined by Student’s *t* test. ns, not significant.


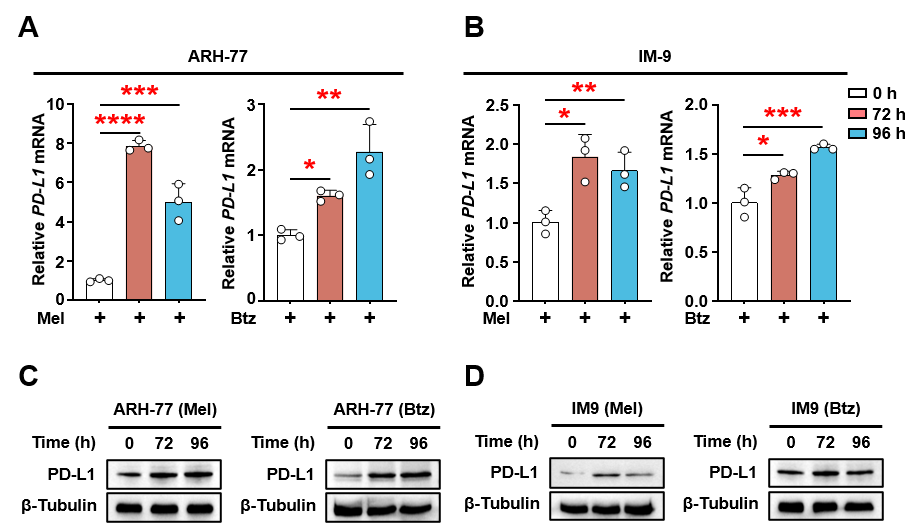


**Figure S3. Melphalan or bortezomib treatment of myeloma cells for an extended period can still upregulate the expression of PD-L1.**

(**A**-**D**) Relative mRNA (**A**, **B**) and protein (**C**, **D**) expression of PD-L1 in ARH-77 or IM-9 cells after melphalan (5 μM) or bortezomib (2.5 nM) treatment for 72 or 96 hours. Data are averages ± SD. Each experiment was repeated three times. **p* < 0.05, ***p* < 0.01, ****p* < 0.001, *****p* < 0.0001. *p* values were determined using one way ANOVA.


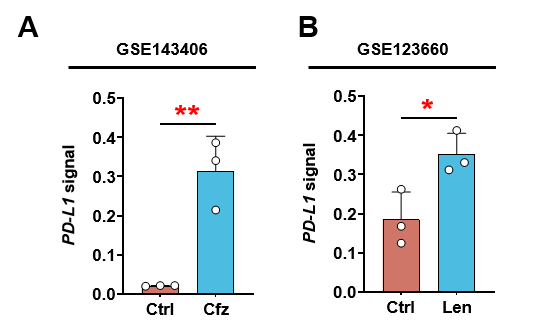


**Figure S4. Carfilzomib or lenalidomide promotes the expression of PD-L1 in myeloma cells.**

(**A**) *PD-L1* mRNA levels of the ARP-1 cells treated with or without carfilzomib (Cfz) (GSE143406) (n = 3 biological replicates). (**B**) *PD-L1* mRNA levels of the OPM-1 cells treated with or without lenalidomide (Len) (GSE123660) (n = 3 biological replicates). **p* < 0.05, ***p* < 0.01. All *p* values were determined by Student’s *t* test.

**
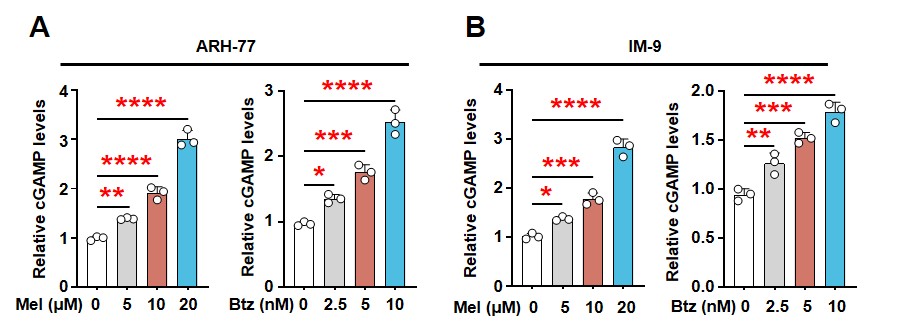
**

**Figure S5. Melphalan or bortezomib enhances the enzymatic activity of cGAS.**

(**A**, **B**) Quantification of cyclic GMP-AMP (cGAMP) by ELISA from cell lysates of ARH-77 or IM-9 cells treated with or without melphalan or bortezomib (n = 3 biological replicates). Data are averages ± SD. **p* < 0.05, ***p* < 0.01, ****p* < 0.001, *****p* < 0.0001. All *p* values were determined using one way ANOVA.

**
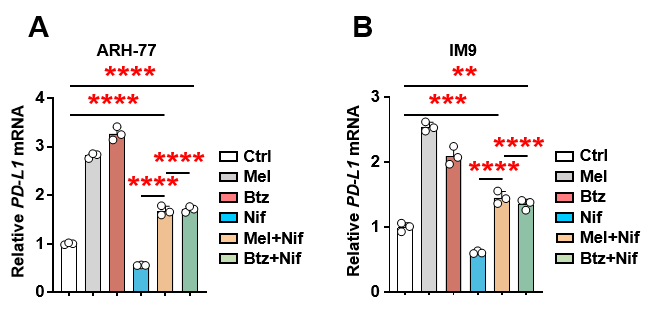
**

**Figure S6. Melphalan or bortezomib promotes PD-L1 expression independent of the STAT pathway.**

(**A**, **B**) The relative expression of *PD-L1* in ARH-77 or IM-9 cells treated with or without melphalan (20 μM), bortezomib (10 nM) or STAT pathway inhibitor (Nifuroxazide, 20 μM) (n = 3 biological replicates). Data are averages ± SD. ***p* < 0.01, ****p* < 0.001, *****p* < 0.0001. All *p* values were determined using one way ANOVA.


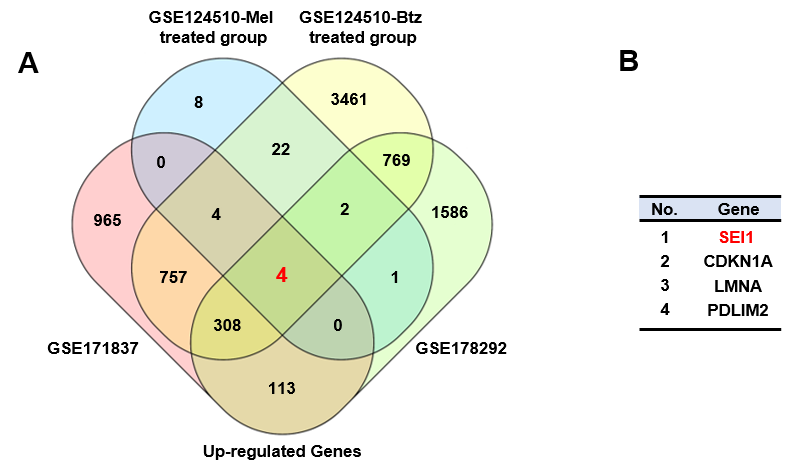


**Figure S7. Venn diagram of differentially expressed genes.**

(**A**, **B**) RNA-seq analysis of Venn diagram of differentially expressed genes, and found 4 genes upregulation in three public RNA-seq datasets (GSE171837, GSE124510 and GSE178292) (n = 3 biological replicates).


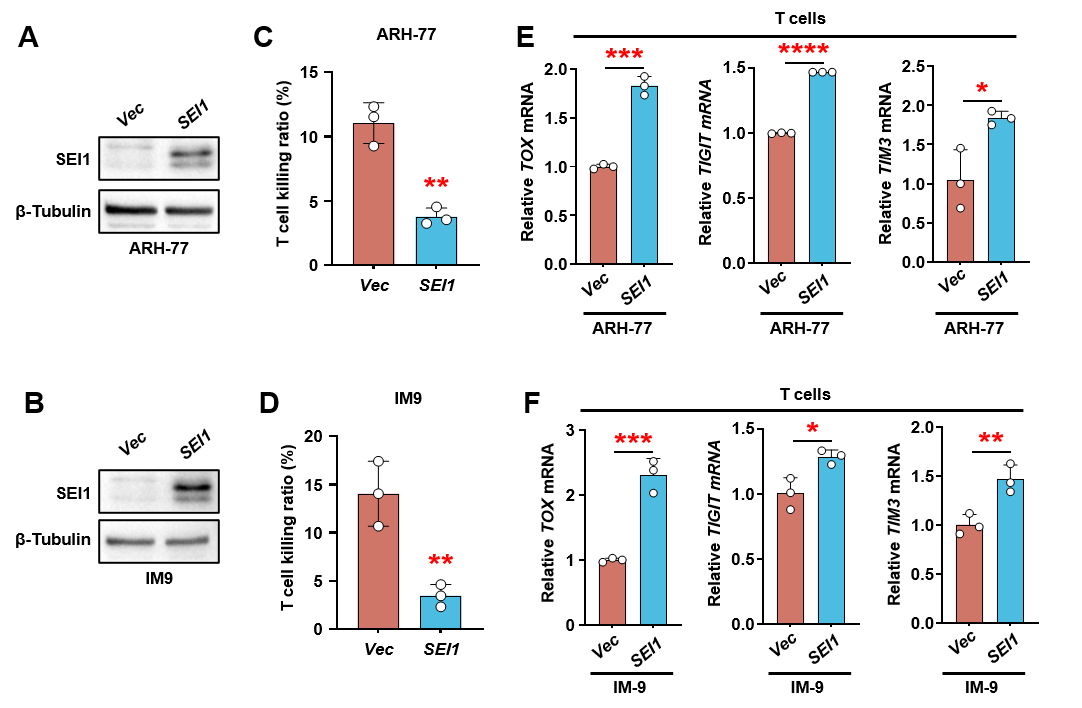


**Figure S8. Myeloma cell SEI1 inhibits the killing efficiency of T cells and induces T cell exhaustion.**

(**A**, **B**) Western blot analysis shows the overexpression of LAMP5 protein in myeloma cells (ARH-77 or IM-9) transfected with the SEI1-overexpressing plasmid as compared to cells transfected with vector control. Data are representative of triplicate blots. (**C**, **D**) T cell-mediated cancer cell killing assay. Myeloma cell lines ARH-77 or MM.1S (*Vec*, *SEI1*) were cocultured with activated T cells for 24 hours were subjected to flow cytometric analysis (n = 3 biological replicates). (**E**, **F**) The relative expression of *TOX*, *TIGIT*, and *TIM3* expression in activated T cells after coculture with myeloma cells (n = 3 biological replicates). Data are averages ± SD. **p* < 0.05, ***p* < 0.01, ****p* < 0.001, *****p* < 0.0001. *p* values were determined by Student’s *t* test.


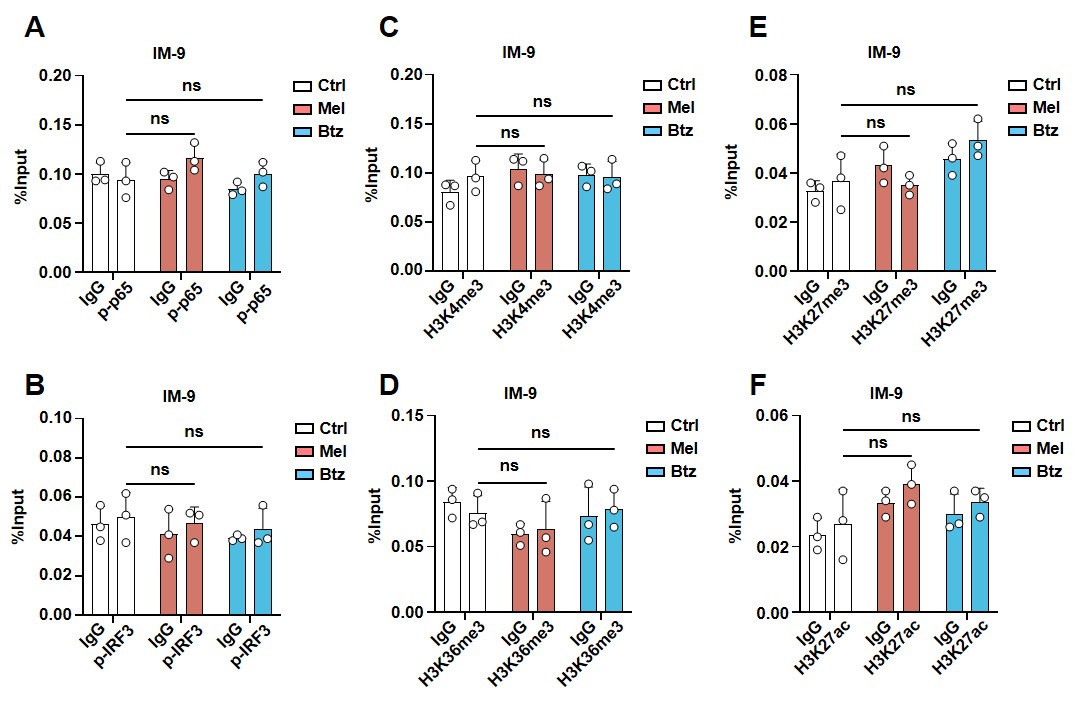


**Figure S9. Melphalan or bortezomib promotes *SEI1* transcription without involving the phosphorylation of p65, IRF3, or histone methylation and acetylation modifications.**

ChIP PCR assay showing phosphor-p65 (**A**), phosphor-IRF3 (**B**), H3K4me3 (**C**), H3K36me3 (**D**), H3K27me3 (**E**), or H3K27ac (**F**) enrichment on *SEI1* promoter of IM-9 cells treated with or without melphalan or bortezomib (n = 3 biological replicates). Data are averages ± SD. All *p* values were determined using one way ANOVA. ns, not significant.


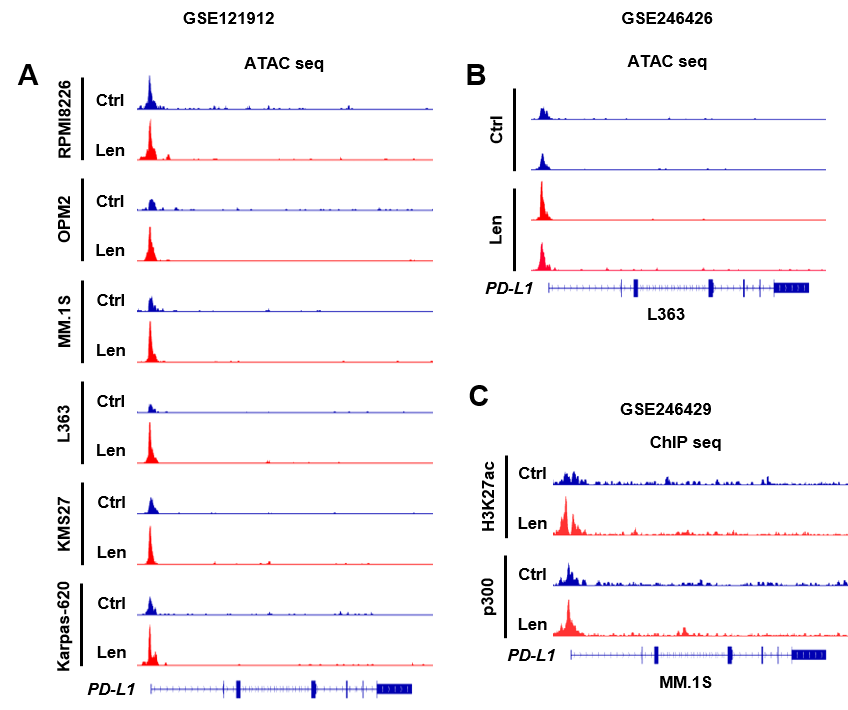


**Figure S10. ATAC-seq and ChIP-seq profiles at *PD-L1* gene loci in myeloma cell lines following treatment with lenalidomide.**

(**A**, **B**) Gene tracks showing representative ATAC-Seq profiles at *PD-L1* gene loci in myeloma cell lines (RPMI8226, OPM2, MM.1S, L363, KMS27 and Karpas-620) following treatment with lenalidomide (GSE121912 and GSE246426). (**C**) ChIP-seq profiles show H3K27ac and p300 signal tracks at the *PD-L1* gene loci for MM.1S cells treated with or without lenalidomide (GSE246429).


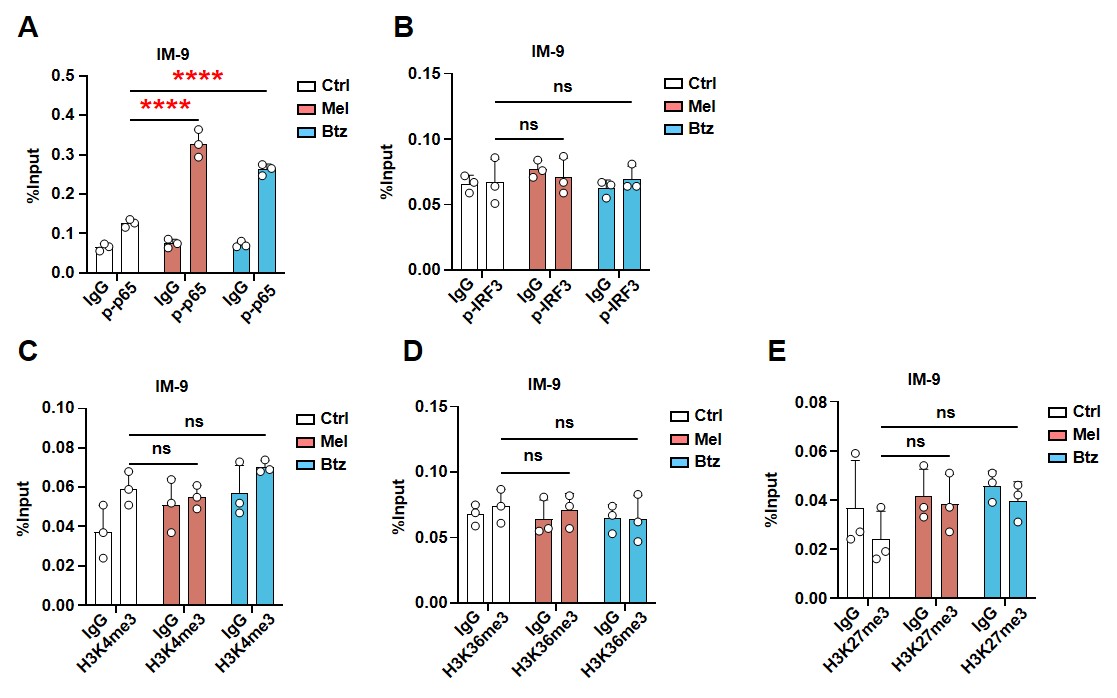


**Figure S11. Melphalan or bortezomib can also promote *PD-L1* transcription through the phosphorylation of p65, but not via the phosphorylation of IRF3 or histone methylation.**

ChIP PCR assay showing phosphor-p65 (**A**), phosphor-IRF3 (**B**), H3K4me3 (**C**), H3K36me3 (**D**), or H3K27me3 (**E**) enrichment on *PD-L1* promoter of IM-9 cells treated with or without melphalan or bortezomib (n = 3 biological replicates). Data are averages ± SD. *****p* < 0.0001. All *p* values were determined using one way ANOVA. ns, not significant.


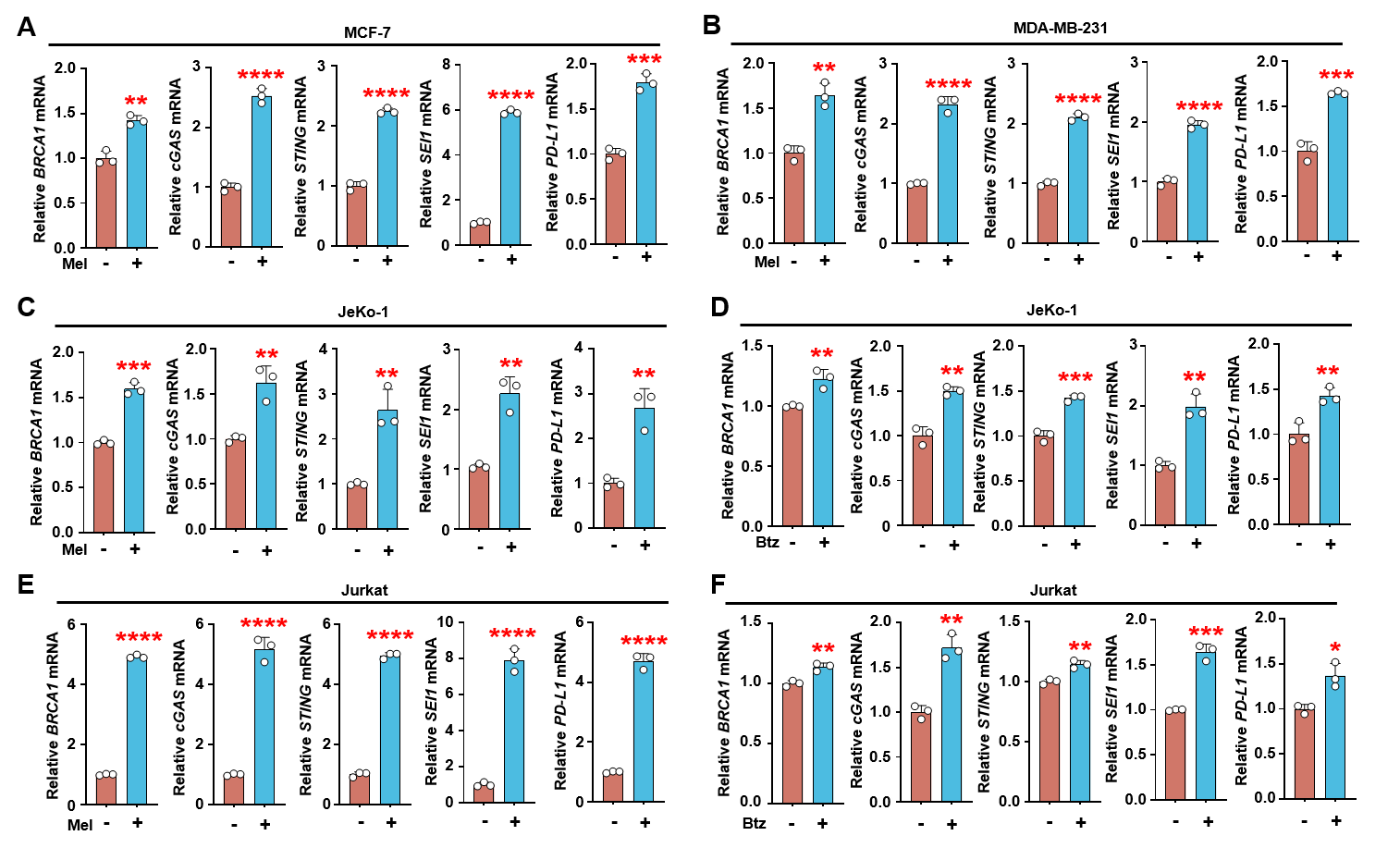


**Figure S12. Melphalan or bortezomib activates the DNA damage/cGAS-STING/**

**SEI1/PD-L1 axis in breast cancer or lymphoma.**

(**A**, **B**) The relative expression of *BRCA1*, *cGAS*, *STING*, *SEI1* or *PD-L1* expression in breast cancer cell lines (MCF-7 or MDA-MB-231) treated with melphalan (n = 3 biological replicates). (**C**-**F**) The relative expression of *BRCA1*, *cGAS*, *STING*, *SEI1* or *PD-L1* expression in lymphoma cell lines (JeKo-1 or Jurkat) treated with melphalan or bortezomib (n = 3 biological replicates). Data are averages ± SD. **p* < 0.05, ***p* < 0.01, ****p* < 0.001, *****p* < 0.0001. All *p* values were determined by Student’s *t* test.


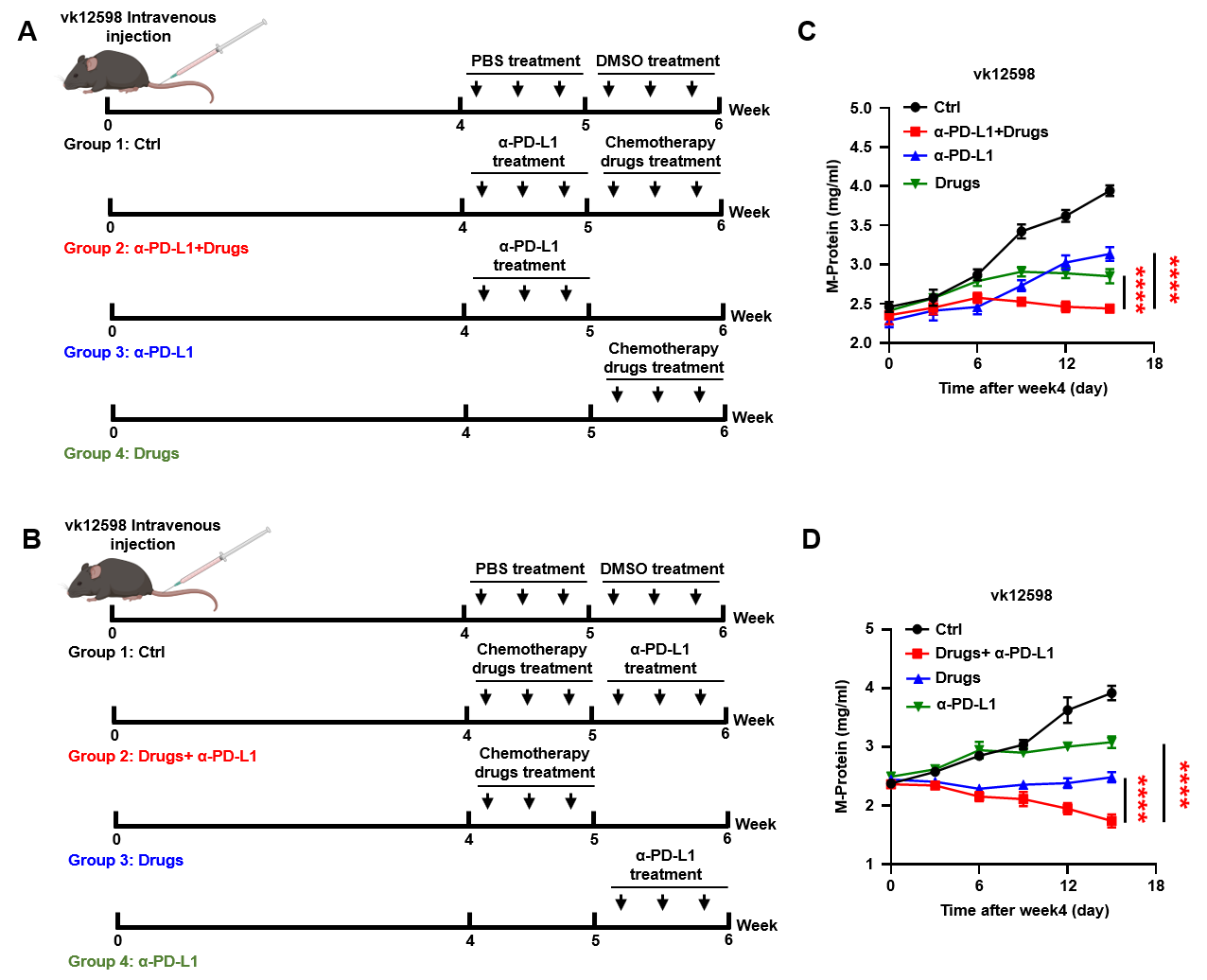


**Figure S13. Comparative efficacy of combination therapy with chemotherapy and PD-L1 antibodies versus monotherapy in myeloma mouse model.**

6-week-old male C57BL/6J mice were intravenously injected vk12598 cells (n = 3 mice/group), followed by intraperitoneal administration of bortezomib (0.5 mg/kg bodyweight) and melphalan (0.5 mg/kg bodyweight) or PD-L1 antibodies (10 mg/kg bodyweight) in different sequences three times for one week each (**A**, **B**). (**C**, **D**) ELISA analysis shown the concentrations of M-protein in mouse sera. Data are averages ± SD. *****p* < 0.0001. *p* values were determined using two-way ANOVA.
